# Nonparametric Risk and Nonparametric Odds in Quantitative Genetic Association Studies

**DOI:** 10.1038/srep12105

**Published:** 2015-07-15

**Authors:** Wei Zhang, Qizhai Li

**Affiliations:** 1 Key Laboratory of Systems Control, Academy of Mathematics and Systems Science, Chinese Academy of Sciences, Beijing 100190, China

## Abstract

The coefficient in a linear regression model is commonly employed to evaluate the genetic effect of a single nucleotide polymorphism associated with a quantitative trait under the assumption that the trait value follows a normal distribution or is appropriately normally distributed after a certain transformation. When this assumption is violated, the distribution-free tests are preferred. In this work, we propose the nonparametric risk (NR) and nonparametric odds (NO), obtain the asymptotic normal distribution of estimated NR and then construct the confidence intervals. We also define the genetic models using NR, construct the test statistic under a given genetic model and a robust test, which are free of the genetic uncertainty. Simulation studies show that the proposed confidence intervals have satisfactory cover probabilities and the proposed test can control the type I error rates and is more powerful than the exiting ones under most of the considered scenarios. Application to gene of PTPN22 and genomic region of 6p21.33 from the Genetic Analysis Workshop 16 for association with the anticyclic citrullinated protein antibody further show their performances.

In binary-trait genetic association studies, the relative risk (RR) is commonly employed to show the degree of risk of the genetic variants associated with human complex diseases. The RR has two important features that it compares the probability of an event occurring in an exposed group to that of the event occurring in a non-exposed group and the reference group ensures the proper comparison. As an alternative, the coefficient in a linear regression model can be reported to show the association strength between the genetic variant and the quantitative trait. When the trait values follow the normal distribution or are normally distributed after a certain transformation such as the Box-Cox transformation, the corresponding statistical analyses are valid. However, the assumption of normality is often violated in practice. For example, for the anticyclic citrullinated protein antibody (anti-CCP) to be analyzed later, neither the observed values nor their logarithm transformation versions satisfy the normal assumption due to truncation. Sometimes, even though some transformations can be used, different transformations might result in different conclusions.

The Kruskal-Wallis test^1^, the Jonckheere-Terpstra test^2^,^3^, the U-statistics-based tests^4^,^5^, and the nonparametric trend test^6^ can be used to evaluate the association between the genetic variants and the quantitative traits for the non-normal trait values. The distributions or the approximate distributions of the test statistics are derived under the null hypothesis that the genotypes are not associated with the traits. However, there is no genetic effect size defined. Recently, Konietschke *et al.*^7^ employed the relative effect 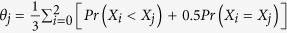 size to measure the genetic effect, where *X*_*j*_ denotes a random variable taking the phenotype values corresponding to genotype *j*, *j* = 0, 1, 2. Based on *θ*_*j*_, they constructed the maximal test considering three genetic models: recessive, additive and dominant models. Also, Brunner and Munzel^8^ defined it as relative treatment effect in solving Behrens-fisher problem and Ryu^9^ called it ordinal effect size measure for the ordered categorical data. However, they did not study the performances of the estimators for *θ*_*j*_ and their confidence intervals, especially under the alternative hypothesis that the genetic variant is associated with the trait, which is of great interest to investigators when a positive finding has been found.

In this paper, we define the nonparametric odds (NO) as λ_*i*_ = Pr(*Y*_0_ < *Y*_*i*_)/[1 − Pr(*Y*_0_ < *Y*_*i*_)], where *Y*_*i*_ denote the trait value that the subjects taking in the group with genotype *i*, *i* = 1, 2. Compared to *θ*_*j*_, *j* = 0, 1, 2, NO has three salient features. First, it gives a comparison of the difference between two groups; the second is that the reference group with genotype value 0 can guarantee a reasonable comparison; third, it equals 1 under the null hypothesis. This paper is organized as follows. In the “Results” section, simulations and real data analysis are conducted to illustrate the performances of the proposed procedures. Some further topics and issues are present in the “Discussion” section. In the “Methods” section, we describe the NO, give its point and confidence interval estimates, and construct the test statistic for a given genetic model and a robust test, which is free of genetic models. At last some technical details are given in the Supplementary Material.

## Results

### Simulation Settings

We conduct simulation studies to explore the performances of the proposed procedures. Consider the linear model 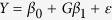, where 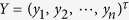 denotes the trait value, 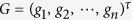 denotes the genotype value at a single nucleotide polymorphism (SNP) locus with 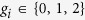 being the count of a certain allele, and 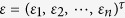 denotes the random error with 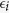 *i.i.d.* following a truncated generalized extreme value distribution (a heavy-tailed distribution), tGEV(0,0,1,0) with the shape parameter 0, the location parameter 0, the scale parameter 1 and the truncated point 0. We consider *β*_0_ = 0.5, 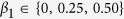 and three minor allele frequencies (MAFs) with 0.15, 0.30 and 0.45. We consider three sample sizes with 500, 1,000 and 1,500, where the results for the sample size of 500 and 1,000 are shown in the Supplemental Material. 2,000 replicates are conducted to calculate the character statistics for each scenario.

### Biases, mean squared error and confidence Interval

Table 1 shows the empirical bias, the square root of mean square error (sMSE), the cover probability (CP) and the interval length (IL) for 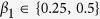 and MAFs 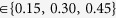. From Table 1, we can find that the proposed three confidence intervals have good cover probabilities with the estimated CP being close to the nominal level of 95%. For example, when *β*_1_ = 0.25 and MAF = 0.15, the cover probabilities of the Standard Interval, the Wilson Interval and the Log-Delta Interval for λ_1_ are 95.3%, 95.1% and 95.1%, respectively, which is very close to the nominal level of 95%. When *β*_1_ = 0.5 and MAF = 0.30, the cover probabilities of the the Standard Interval, the Wilson Interval and the Log-Delta Interval for λ_2_ are 94.6%, 94.9% and 94.9%, respectively. The Wilson Interval has the smallest IL and the standard interval have the largest IL among them.

Table 2 show the results of empirical biases, sMSE, CP and IL of the proposed procedures under the recessive, additive and dominant models. Similar findings are obtained to those from Table 1. All the three confidence interval estimation procedures can keep the cover probabilities correctly and the IL of the Wilson Interval is the shortest among the three confidence intervals. The Standard Interval usually gives large IL. For example when MAF = 0.15, *β*_1_ = 0.25 and the genetic model is recessive, the empirical CP of the Standard Interval, the Wilson Interval and the Log-Delta Interval are equal to 94.2%, 94.8% and 94.9%, respectively, and the IL of the Wilson interval is (1.313), which is smaller than those of the Standard Interval (1.363) and the Log-Delta Interval (1.319). In general, all the three confidence interval estimation procedures can be used in practice. If we want to choose one, the Wilson Interval is recommended.

### Type I error rates

We first evaluate the empirical type I error rates of the the proposed *Z*_*R*_, *Z*_*A*_ and *Z*_*D*_ compared with the exiting Kruskal-Wallis test, F test and Jonckheere-Terpstra test under a given genetic model. To make a fair comparison, we construct the Kruskal-Wallis, F and Jonckheere-Terpstra tests under thee genetic models separately. For the simplicity of notations, we denote the Kruskal-Wallis test, F test and Jonckheere-Terpstra test under the recessive model by KW-R, F-R and JT-R, respectively, under the additive model by KW-A, F-A, and JT-A, respectively, and under the dominant model by KW-D, F-D and JT-D, respectively. Besides three MAFs of 0.15, 0.30 and 0.45, we also consider MAF of 0.05. The nominal significance level is set to be 0.05. Table 3 shows the empirical type I error rates. As expected, for MAFs of 0.15, 0.30 and 0.45, all the considered procedures can control type I error rates. For example, when MAF is 0.15, the empirical type I error rates of the KW-R, KW-A, KW-D, F-R, F-A, F-D, JT-R, JT-A, JT-D, *Z*_*R*_, *Z*_*A*_, and *Z*_*D*_ test are 0.043, 0.048, 0.055, 0.041, 0.048, 0.051, 0.035, 0.041, 0.042, 0.041, 0.051, and 0.054, respectively. We next explore the empirical type I error rates of the robust tests: Konietschke *et al.*’s test^7^ (for convenience, denote it by KLH) and MAX3. Table 3 gives the results. It indicates that both KLH and MAX3 can control the type I error rates for MAFs of 0.15, 0.30 and 0.45. For MAF of 0.05, KLH is too optimistic with the empirical type I error rates 0.174, which is by far larger than 0.05. We then consider a stringent nominal level of 5 × 10^−4^. Similar results are observed, see Table S19 for details in the Supplementary Material. For the F, Jonckheere-Terpstra and KLH procedures, We use the existing R-software package to calculate the corresponding p-values. Specifically, the function *lm* in the package *stats* to compute the statistical significance of the F test, the function *jonckheere.test* in the package *clinfun* to compute that of the Jonckheere-Terpstra test, and the function *nparcomp* in the package *nparcomp* to compute that of the KLH test.

### Power Comparison

**Figure 1:**
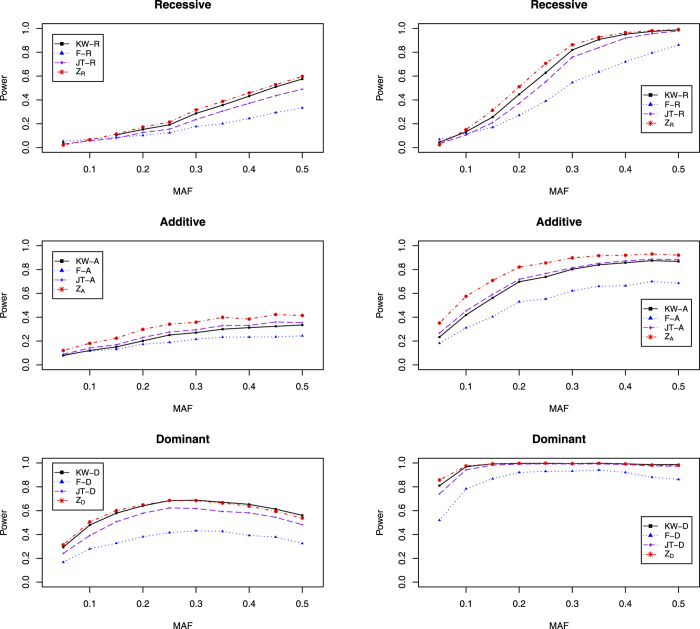
The empirical power of the Kruskal-Wallis test (KW-R, KW-A and KW-D), the Jonckheere-Terpstra test (JT-R, JT-A and JT-D), the F test (F-R, F-A and F-D) and the proposed nonparametric test (*Z*_*R*_, *Z*_*A*_ and *Z*_*D*_) derived under a given genetic model. The first column is for *β*_1_ = 0.25 and the second column is for *β*_1_ = 0.5.

We conduct simulation studies to compare the power among the above tests. We use the same simulation settings as above and set *β*_0_ = 0.50, 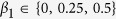 and the error term *ε* ~ tGEV(0, 0, 5, 0). *n* = 1,500 and 2,000 replicates are conducted to calculate the power. The nominal significance level is 0.05. Figure 1 shows the empirical power of *Z*_*R*_, *Z*_*A*_, *Z*_*D*_, KW-R, KW-A, KW-D, F-R, F-A, F-D, JT-R, JT-A, and JT-D. It indicates that the proposed tests is more powerful than the other tests, especially under the recessive and additive model, sometimes there is more than 10% power increase. For example, when the MAF is 0.10, *β*_1_ = 0.50 and the genetic model is additive, the empirical powers of *Z*_*A*_, KW-A, F-A, and JT-A are 0.585, 0.419, 0.311, and 0.453, respectively, and when the MAF is 0.25, *β*_1_ = 0.50 and the genetic model is recessive, the empirical powers of *Z*_*R*_, KW-R, F-R, and JT-R are 0.717, 0.618, 0.390, and 0.552, respectively.

**Figure 2:**
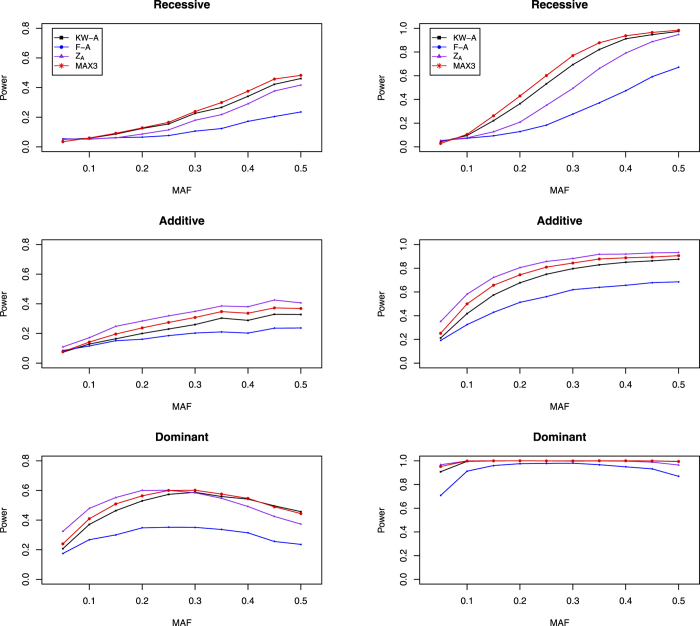
The empirical power of KW-A, F-A, *Z*_*A*_ and MAX3. The first column is for *β*_1_ = 0.25 and the second column is for *β*_1_ = 0.5.

Figure 2 shows the empirical power of four tests, KW-A, F-A, *Z*_*A*_ and MAX3. Since for MAF of 0.05 and that being less than 0.15, KLH cannot control the type I error rate under the nominal significance level of 0.05 and 5 × 10^−4^, respectively, we do not include it here to make a fair comparison. From Figure 2, it can be seen that MAX3 performs more powerful than the other compared test under most of scenarios, especially when the genetic models are recessive and dominant models. For example, when MAF = 0.30, *β*_1_ = 0.5, and the genetic model is recessive, the powers of the KW-A, F-A, *Z*_*A*_, and MAX3 are 0.693, 0.276, 0.493, and 0.770, respectively. Under the additive model, *Z*_*A*_ performs the best among them, it is reasonable since the data are generated under it. So, MAX3 is the most robust test among them.

### Applications to the gene PTPN22 and the genomic region of 6p21.33

It is well known that the genetic variants are deleterious to rheumatoid arthritis (RA)^10^. The anti-CCP taking continuous values, are much more common in the blood of individuals with RA than those without it^11^. The specificity using the anti-CPP to diagnose RA lies between 87.8% and 96.4%^12^. The gene PTPN22 and genomic region of 6p21.33 were reported to be associated with RA^13^,^14^. We apply the proposed procedures to the gene PTPN22 data including 25 SNPs and the genomic region of 6p21.33 including 45 SNPs from the Genetic Analysis Workshop 16^15^. The data consists of 868 cases and 1,194 controls, where 868 subjects have the anti-CCP measure and 1,194 do not have them. The minimum value of anti-CCP for these 868 individuals is 20.053. As shown in Li *et al.*^6^, neither the original values of anti-CCP (p-value using Shapiro-Wilk test is 10^−16^) nor their logarithm transformation ones (p-value is 3.9 × 10^−9^) follow the normal distribution. It is most likely that the observed anti-CCP measurements come from a truncated distribution since the anti-CCP is often measured to confirm RA and so it is not measured in controls. So the statistical analysis under the normal assumption is not reasonable.

Here we only use the genotype data in cases since the anti-CCP values are missing in controls. Table S20 in the Supplemental Material shows the p-values of 25 SNPs in gene PTPN22 and Table S21 shows the p-values of 45 SNPs in the genomic region of 6p21.22 using the KW-A, F-A, *Z*_*A*_, and MAX3. To evaluate the accuracy of the derived formulas, we also include 100,000 permutations to calculate the p-value of MAX3 in the table. The p-value results using permutation is very close to that derived from the asymptotic distribution. We find that the minimum p-value for 25 SNPs is 0.018 using MAX3. Under the significance level of 0.05, and after the Bonferroni correction, no significance reveals. The reason might be that we do not use the data whose anti-CCP measures are less than 20.053. For the genomic region of 6p21.22, there is one SNP rs2734583, which is significant under the significance level of 0.05, and the Bonferroni correction. The p-values using KW-A, F-A, *Z*_*A*_ and MAX, are 0.0004, 0.0009, 0.0014, and 0.0008, respectively.

## Discussions

Linear regression model is a classical approach to evaluate the association between the genetic variants and the continuous traits, where the coefficient in the model indicates the magnitude of the risk of a candidate SNP. When the trait values are normally distributed, most statistical analysis techniques are available. When it is violated, although the linear model can still be done, the estimated coefficient can not measure the real strength of the deleterious SNP. The log-transformation or the Box-Cox transformation is often employed to handle the non-normal quantitative trait. However, different transformations might result in different conclusions. In this work, we define the NR and NO, and give their point and confidence interval estimates under the general framework. Then we define the recessive, additive and dominant genetic models using NO and propose the corresponding point and confidence interval estimates. We also provide three test statistics under different genetic models and a robust test being free of genetic model. Extensive simulation studies shows that the cover probabilities of the proposed procedures are satisfactory, because they are close to the nominal level, and the proposed test is powerful than the exiting procedures under a given genetic model. Applications to GAW16 data for anti-CCP further show the performances of the proposed procedures.

There are several issues that can be further investigated. First in this work we study only the scenario for the common allele under the hypothesis of common disease common allele. Under the hypothesis of common disease rare allele, how to extend the proposed procedure to this situation is worth considering, where the large sample theory does not hold. Secondly, we focus on the biallelic marker, the proposed procedures can be readily extended to multiallelic locus, and we can similarly define the NR and NO and construct the confidence intervals of NO. Thirdly, it is well known that family-based association studies is less susceptible to the confounding factors than population-based studies, so a trio design with two parents and an offspring could be considered in the future.

Confounding factors can not be ignored and adjusting for covariates is an important issue in population-based genetic association studies. For example, The hidden population structure can lead to many false-positive findings and correcting for population stratification has become a routine in genome-wide association studies (GWASs)^16^,^17^. To remove the effect of the covariates, we can use the same strategies as EIGENSTRAT^16^. We first compute the residuals of the trait after regressing out the covariates and then take the residuals as the new outcome rather than the original trait values. Simulation results shown in the Supplemental Material are consistent with those without considering the covariates.

In the proposed NR and NO, we do not consider the ties among the observations because the probability of the event that there are ties in the observations for the phenotype taking continuous values is zero. However, if there are ties among the sample, we can add 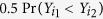 with 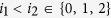 to the formulas of NR and NO, and 0.5 × (number of ties between group with genotype *i*_1_ and group with genotype *i*_2_)/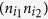 to the estimators.

To calculate λ_1*A*_ under the additive model, the linear combination of 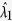 and 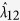 is used. Here, we adopt the proportion of sample size for each NO estimate over the total sample sizes as the weights. There are also some other available weights. For example, the MSE-based weights proposed by Zhong and Prentice^18^ to reduce the bias of log-odd ratios in a two-stage GWAS, and the square root of the sample proportion given by^19^ to jointly analyze the test statistics in a two-stage GWAS. So a comparison of the efficiency of these weights is a valued topic.

In the Supplementary Material, we prove that both 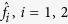 and 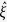 follow the standard normal distribution as min{*n*_0_,*n*_1_,*n*_2_} goes to infinity. However, how many samples we need to get an accurate estimation? Based on the simulations, 1,000 more sample size is needed to get a reasonable estimate. In the current GWASs, the sample size is tens of thousands. For example, the Wellcome Trust Case Control Consortium (2007)^20^ used about 2,000 cases for each of seven diseases and 3,000 shared controls to detect the deleterious SNPs. Another example was Landi *et al.*’s (2009)^21^ Lung GWAS, where 5,939 cases and 5,848 controls were genotyped to identify the hereditary contribution to adenocarcinoma.

In the simulations, we consider the situation where the MAF is greater than 0.1, the sample size is larger than 1,000 and the distribution is truncated generalized extreme value distribution. Actually, we also conduct the simulation studies considering that MAF is 0.05, the sample size is 1000 and 500, and the distribution is normal distribution and centralized *t* distribution. Results given in the Supplemental Material indicate that the proposed test is more powerful than the existing ones under most of the considered scenarios.

The proposed NR and NO can estimate the non-parametric disease risk and odds in studies with “prospective sampling” such as a cohort study. However, in case-control studies, the case/control ratio for recruited subjects is different from the true one in the population. The derived tests can still be used to detect the association between the quantitative trait and genetic variants in case-control association studies. Finally, the proposed procedure has been coded in R verion 3.1.1 and is freely requested from the corresponding author.

## Methods

### NR and NO

Suppose that *n* subjects are enrolled from a source population in a quantitative trait genetic association study. A biallelic SNP is considered here. Assume that the genotype is coded as 0, 1, and 2, corresponding to the count of a certain allele. Let 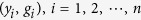 be the observed sample, where *y*_*i*_ and *g*_*i*_ denote the trait value and genotype value of the *i*th subject. For the convenience of notations, let the first *n*_0_ subjects have the genotype 0, the second *n*_1_ subjects have the genotype 1, and the last *n*_2_ subjects possess the genotype 2. Suppose that the group with genotype 0 is the reference group. Denote *f*_*i*_ = Pr(*Y*_0_ < *Y*_*i*_), where *Y*_0_, *Y*_1_ and *Y*_2_ denote the random variables that take the values in three sets 
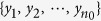
, 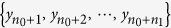, and 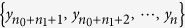, respectively. Here we do not consider that there exit ties in the sample because the probability of the ties between two genotype groups is zero when the random variables *Y*_0_,*Y*_1_ and *Y*_2_ are continuous. *f*_*i*_ shows the probability that the trait values in a group with genotype *i* is stochastically larger than those in the reference group with gentoype 0, *i* = 1,2. We call *f*_1_ and *f*_2_ the nonparametric risk (NR). Then we define the NO as λ_*i*_ = *f*_*i*_/(1 − *f*_*i*_), *i* = 1, 2. We point out that when any of the *f*_1_ or *f*_2_ is one, the estimator for λ_1_ or λ_2_ is not defined, although this is unlikely to occur in practice. The null hypothesis is *H*_0_ : *f*_1_ = *f*_2_ = 0.5 or λ_1_ = λ_2_ = 1. The alternative hypothesis is *H*_1_ : *f*_2_ ≥ *f*_1_ ≥ 0.5 and *f*_2_ > 0.5 or equivalently, λ_2_ ≥ λ_1_ ≥ 1 and λ_2_ > 1.

The empirical estimators of *f*_1_ and *f*_2_ are, respectively,


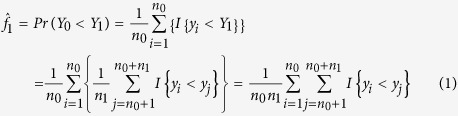


and


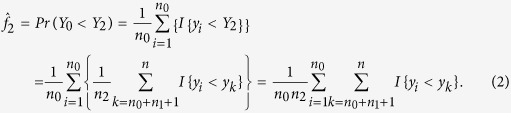


The estimators of the variances of 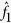 and 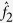 are, respectively,


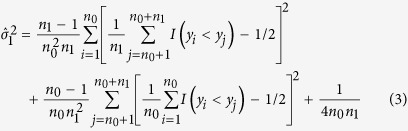


and


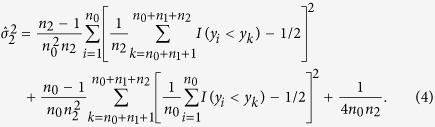


The estimator of the covariance between 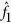 and 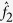 is


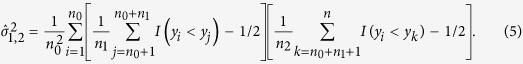


### Confidence interval

From the theorem in the Supplementary Material, we know that 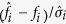 asymptotically follows a standard normal distribution. So the confidence intervals of *f*_*i*_ at the significance level of 1 − *α* (0 < *α* < 1) is


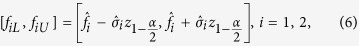


where 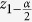 is the 1 − *α*/2 quantile of the standard normal distribution. Since λ_*i*_ is a strictly monotonic increasing function of *f*_*i*_, the Standard Interval of λ_*i*_ is


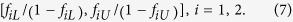


Besides the Standard Interval, we can also construct the Wilson Interval^22^,^23^. 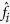 can be thought to estimate the success probability of a binomial distribution with two parameters (*N*_*i*_, *f*_*i*_) where *N*_*i*_ represent the number of trials and *f*_*i*_ is the success probability. We estimate the efficient *N*_*i*_ by setting 
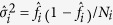
. Then the number of successes is 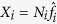, *i* = 1, 2. So, the Wilson Interval has the form of 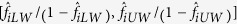, where


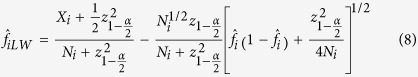


and


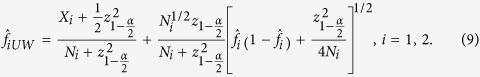


The Log-Delta interval is obtained by using the delta method on the logarithm of λ_*i*_, *i* = 1, 2. Denote 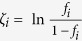 and 
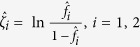
. Based on the result of the theorem in the Supplementary Material and the delta method, we have


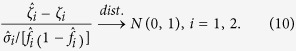


Then the confidence intervals of *ζ*_*i*_ at the significance level of 1 − *α* is


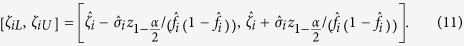


Since λ_*i*_ = exp(*ζ*_*i*_) is a strictly monotonic increasing function of *ζ*_*i*_, the Log-Delta interval of λ_*i*_ is
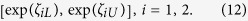


### Considering Genetic Models

A genetic model refers to a specific mode of inheritance. The commonly used genetic models in binary trait genetic association studies are recessive, additive and dominant models. For the non-normal distributed quantitative trait, the genetic models and the corresponding point estimates of NO are given in Table 4, where we define the additive model in the scale of NO as λ_1_ = λ_12_ > 1 and λ_2_ > 1 with λ_12_ = *f*_12_/(1 − *f*_12_) and *f*_12_ = Pr(*Y*_1_ < *Y*_2_). In other words, the odds of trait values in the group with genotype 2 relative to those in group with genotype 1 is equal to that of trait values in group with genotype 1 relative to those in group with genotype 0. Based on the definitions, λ_1_ = λ_12_ is equivalent to *f*_1_ = *f*_12_.

Using the notations in Table 4, we substitute 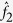 by 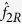 and 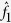 by 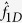 and the corresponding three confidence intervals for *f*_1*R*_ and *f*_1*D*_ can be obtained. The construction of the confidence interval for λ_2*A*_ under the additive model is similar to that of 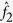. We now derive the confidence interval of 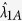. Denote 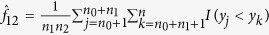, From the theorem in the Supplementary Material, we know that 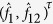 asymptotically follows a bivariate normal distribution with mean vector 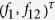 and covariance matrix Δ. The consistent estimate of Δ is


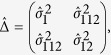


where


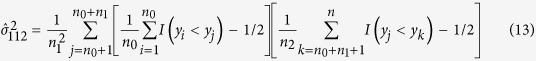


and


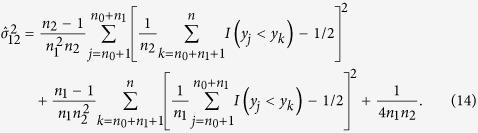


Thus the variance of 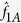 is


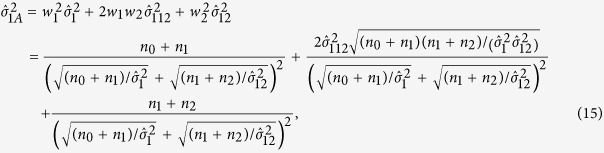


where 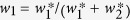, 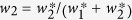, 
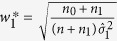
 and 
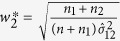
. Under *H*_0_, 
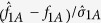
 asymptotically follows a standard normal distribution. Similar to the above procedures, we can construct the Standard Interval, the Wilson Interval and the Log-Delta Interval.

### Nonparametric Test Statistic under a given genetic model

We construct the nonparametric test statistics under the recessive, additive and dominant models to test *H*_0_. The test statistic under the recessive model is 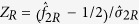, where


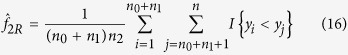


and


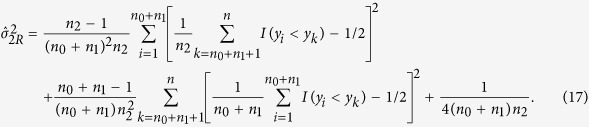


Under *H*_0_, 
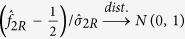
. So the two-sided p-value is


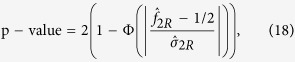


where 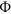 (.) denotes the cumulative distribution function of the standard normal distribution.

Under the additive model, the test statistic is 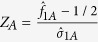, 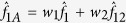, and


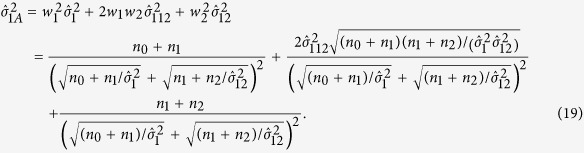


So the two-sided p-value is


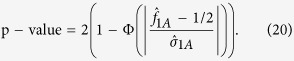


Similar to the recessive model, the test statistic under the dominant model is 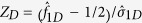. The definition of 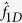 is given in Table 4, and


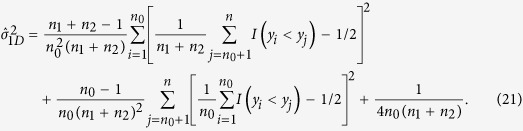


Under *H*_0_, 
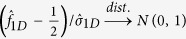
. So the two-sided p-value is


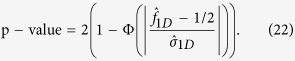


### MAX3

In the above section, we define λ_1_ and λ_2_ and give some asymptotical statistical properties. However, they do not consider the genetic model. The test statistic *Z*_*A*_ is obtained by combining the data of subjects with genotype 0 and 1, and *Z*_*D*_ is obtained by combining the data of subjects with genotype 1 and 2. *Z*_*A*_ is obtained by weighting λ_1_ and λ_2_. They can be seen the combination of λ_1_ and λ_2_ to some extent under the fixed genetic model. When the genetic model is fixed for a given SNP, one can use the above tests to do the association studies. However, the mode of genetic inheritance is often unknown in practice. The test obtained under an improper model might lose power substantially. So, a robust test, which is free of genetic model is preferred. Let’s consider a robust test


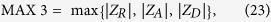


the maximum value of three nonparametric tests. Under *H*_0_, (*Z*_*R*_, *Z*_*A*_, *Z*_*D*_)^*τ*^ follows a three dimensional normal distribution^24^ with mean (0, 0, 0) and covariance matrix 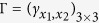, 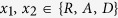, where 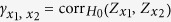 is the covariance of the statistics 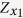 and 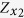 under the null hypothesis, which is given in the Supplementary Material. The detailed derivations of *γ*_*RA*_,* γ*_*AD*_ and *γ*_*RD*_ are also presented in the Supplementary Material. Then for the observed value of MAX3, we use the function *pmvnorm* in R pacakge *mvtnorm* to calculate its p-value.

## Additional Information

**How to cite this article**: Zhang, W. and Li, Q. Nonparametric Risk and Nonparametric Odds in Quantitative Genetic Association Studies. *Sci. Rep.* **5**, 12105; doi: 10.1038/srep12105 (2015).

## Supplementary Material

Supplementary Information

## Figures and Tables

**Table 1:** The empirical bias, sMSE, CP and IL of NO.

MAF	Bias	sMSE	Standard Interval		Wilson Interval		Log-Delta Interval	
			CP	IL	CP	IL	CP	IL
**Inferring λ**_**1**_**(=1.43) under** ***β***_**1**_ **=** **0.25**								
0.15	−0.0085	0.092	0.953	0.369	0.951	0.367	0.951	0.367
0.30	0.0384	0.096	0.936	0.354	0.939	0.353	0.939	0.353
0.45	−0.0052	0.101	0.948	0.404	0.947	0.402	0.948	0.402
**Inferring λ**_**2**_**(=1.97) under** ***β***_**1**_** = 0.25**								
0.15	0.0160	0.350	0.932	1.382	0.932	1.329	0.932	1.336
0.30	−0.0038	0.184	0.953	0.757	0.953	0.748	0.953	0.749
0.45	−0.0200	0.168	0.951	0.677	0.949	0.671	0.949	0.672
**Inferring λ**_**1**_**(=1.97) under** ***β***_**1**_** = 0.5**								
0.15	−0.0047	0.126	0.953	0.504	0.952	0.501	0.952	0.501
0.30	0.0072	0.125	0.956	0.506	0.955	0.504	0.955	0.504
0.45	−0.0010	0.154	0.945	0.595	0.944	0.591	0.944	0.591
**Inferring λ**_**2**_**(=3.58) under** ***β***_**1**_** = 0.5**								
0.15	0.0596	0.589	0.940	2.314	0.942	2.204	0.943	2.212
0.30	0.0085	0.350	0.946	1.394	0.949	1.369	0.949	1.371
0.45	0.0982	0.371	0.932	1.379	0.938	1.355	0.938	1.357

**Table 2:** The empirical bias, sMSE, CP and IL of NO under different genetic model.

MAF	Bias	sMSE	Standard Interval		Wilson Interval		Log−Delta Interval	
			CP	IL	CP	IL	CP	IL
**Inferring λ**_**2*****R***_**(=1.97) under the recessive model with** ***β***_**1**_** = 0.25**								
0.15	0.0438	0.342	0.942	1.363	0.948	1.313	0.949	1.319
0.30	0.0092	0.174	0.948	0.701	0.948	0.694	0.948	0.695
0.45	−0.0201	0.135	0.948	0.528	0.945	0.525	0.945	0.525
**Inferring λ**_**2*****R***_**(=3.58) under the recessive model with** ***β***_**1**_** = 0.5**								
0.15	0.0535	0.593	0.933	2.264	0.930	2.160	0.931	2.168
0.30	0.0458	0.314	0.946	1.225	0.946	1.208	0.946	1.210
0.45	0.0088	0.252	0.949	0.989	0.950	0.980	0.950	0.981
**Inferring λ**_**1*****A***_**(=1.43) under the additive model with** ***β***_**1**_** = 0.25**								
0.15	−0.0153	0.080	0.938	0.306	0.938	0.305	0.938	0.305
0.30	−0.0029	0.062	0.954	0.249	0.954	0.249	0.954	0.249
0.45	−0.0018	0.061	0.939	0.239	0.939	0.239	0.939	0.239
**Inferring λ**_**1*****A***_**(=1.97) under the additive model with** ***β***_**1**_** = 0.5**								
0.15	0.0077	0.104	0.943	0.413	0.945	0.412	0.945	0.412
0.30	0.0055	0.089	0.946	0.352	0.945	0.351	0.945	0.351
0.45	−0.0060	0.089	0.949	0.348	0.949	0.347	0.949	0.347
**Inferring λ**_**1*****D***_**(=1.97) under the dominant model with** ***β***_**1**_** = 0.25**								
0.15	−0.0033	0.128	0.935	0.492	0.933	0.490	0.933	0.490
0.30	0.0041	0.123	0.952	0.491	0.953	0.489	0.953	0.489
0.45	0.0028	0.145	0.951	0.578	0.948	0.574	0.948	0.574
**Inferring λ**_**1*****D***_**(=3.58) under the dominant model with** ***β***_**1**_** = 0.5**								
0.15	0.0440	0.243	0.943	0.952	0.947	0.944	0.947	0.945
0.30	0.0434	0.262	0.943	1.020	0.947	1.010	0.947	1.011
0.45	−0.0165	0.321	0.940	1.248	0.937	1.230	0.937	1.232

**Table 3:** The empirical type I error rates of the Kruskal-Wallis test^1^ (KW-R, KW-A, KW-D), the F test (F-R, F-A, F-D), the Jonckheere-Terpstra test^2^,^3^ (JT-R, JT-A, JT-D), KLH^7^and the proposed test (*Z*_*R*_, *Z*_*A*_, *Z*_*D*_) and the proposed MAX3.

MAF	KW-R	KW-A	KW-D	F-R	F-A	F-D	JT-R	JT-A	JT-D	KLH	*Z*_*R*_	*Z*_*A*_	*Z*_*D*_	MAX3
0.05	0.048	0.045	0.054	0.048	0.059	0.059	0.037	0.040	0.044	0.174	0.016	0.055	0.053	0.032
0.15	0.043	0.048	0.055	0.041	0.048	0.051	0.035	0.041	0.042	0.060	0.041	0.051	0.054	0.046
0.30	0.052	0.049	0.047	0.051	0.052	0.054	0.038	0.037	0.037	0.053	0.053	0.049	0.047	0.047
0.45	0.040	0.049	0.047	0.045	0.045	0.045	0.036	0.037	0.037	0.048	0.039	0.051	0.044	0.046

The nominal level is 0.05 and 2,000 replicates are conducted.

**Table 4:**
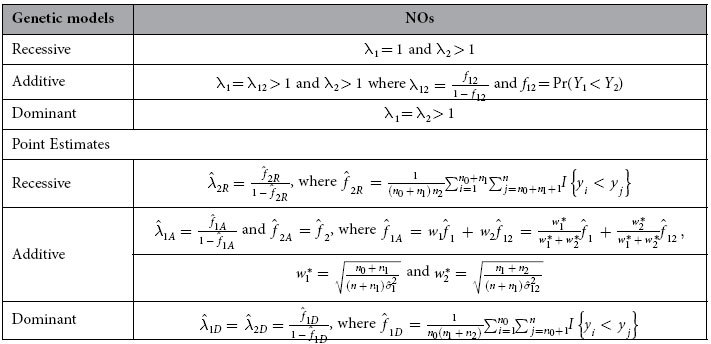
NO and their point estimates under different genetic models.
