# Supplementary material for: Nonparametric Risk and Nonparametric Odds in Quantitative Genetic Association Studies
Source: Sci Rep. 2015 Jul 15;5:12105. doi: 10.1038/srep12105 (PMC5378889; doi:10.1038/srep12105)

Online Supplementary Material for

Nonparametric Risk and Nonparametric Odds in Quantitative Genetic Association Studies

Wei Zhang and Qizhai Li

Contents

1. Some technical details of the obtained statistics.

    1.1. Theorem.

    1.2. Procedure of testing $\lambda_1 = \lambda_{12}$

    1.3. Derivation of estimate for the variance of $\hat{f}_1$ and $\hat{f}_2$.

    1.4. Estimates of the covariances for Z_R, Z_A and Z_D under H_0.

2. Additional simulation results without considering the covariates.

3. Additional simulation results for sample size $n = 1000$ and $n = 500$.

4. Estimation procedures considering the covariates.

5. Simulation studies considering the covariates.

6. The empirical type I error rates under the nominal significance level of 0.0005.

7. P-values of KW-A, F-A, Z_A and MAX3 for 25 SNPs in the gene PTPN22 and 45 SNPs in the genomic region of 6p21.33.

8. Simulation results for the random error term following the normal distribution.

9. Simulation results for the random error term following the centralized t-distribution.

1. Some technical details of the obtained statistics.

1.1. Theorem.

Denote $\xi = \big(f_1, f_{12}\big)^\tau$ and $\hat{\xi} = \big(\hat{f}_1, \hat{f}_{12}\big)^\tau$. When $\min\{n_0, n_1, n_2\} \longrightarrow \infty$,

1) $(\hat{f}_i - f_i)\big/\hat{\sigma}_i \xrightarrow{dist.} N(0,1), i = 1, 2$, and $(\hat{f}_{12} - f_{12})\big/\hat{\sigma}_{12} \xrightarrow{dist.} N(0,1)$.

2) $\hat{\Delta}^{-\frac{1}{2}}(\hat{\xi} - \xi)$ asymptotically follows a bivariate normal distribution with zero mean vector and covariance matrix I_2, where I_2 is a 2×2 identify matrix.

Proof. Since $\hat{f}_i - f_i$ and $\hat{\xi} - \xi$ are U-statistics, they converge to the normal distributions.

1.2. Procedure of testing $\lambda_1 = \lambda_{12}$.

From the above Theorem, we know that $\big(f_1, f_{12}\big)^\tau$ asymptotically follows a bivariate normal distribution. Then we can use the statistic $\frac{f_1 - f_{12}}{\sqrt{\hat{\sigma}_1^2 - 2\hat{\sigma}_{112}^2 + \hat{\sigma}_{12}^2}}$ to test $\lambda_1 = \lambda_{12}$. The two-sided p-value is

$$\text{p-value} = 2\left(1 - \Phi\left(\big|\hat{f}_1 - \hat{f}_{12}\big|\Big/\sqrt{\hat{\sigma}_1^2 - 2\hat{\sigma}_{112}^2 + \hat{\sigma}_{12}^2}\right)\right).$$

1.3. Derivation of estimate for the variance of $\hat{f}_1$ and $\hat{f}_2$.

Since $\hat{f}_1 = \frac{1}{n_0 n_1}\sum_{i=1}^{n_0}\sum_{j=n_0+1}^{n_0+n_1} I(y_i < y_j)$, we have

$$\begin{aligned}
\text{Var}(\hat{f}_1) &= E\left(\frac{1}{n_0 n_1}\sum_{i=1}^{n_0}\sum_{j=n_0+1}^{n_0+n_1} I(y_i < y_j) - f_1\right)^2 \\
&= \frac{1}{n_0^2 n_1^2}\sum_{i_1=1}^{n_0}\sum_{i_2=1}^{n_0}\sum_{j_1=n_0+1}^{n_0+n_1}\sum_{j_2=n_0+1}^{n_0+n_1} E\big[I(y_{i_1} < y_{j_1}) - f_1\big]\big[I(y_{i_2} < y_{j_2}) - f_1\big].
\end{aligned}$$

Define $\xi^{(i_1,i_2,j_1,j_2)} = E\big[I(y_{i_1} < y_{j_1}) - f_1\big]\big[I(y_{i_2} < y_{j_2}) - f_1\big]$, for $1 \le i_1, i_2 \le n_0$, $n_0 + 1 \le j_1, j_2 \le n_0 + n_1$. Then we have

when $i_1 \ne i_2,\ j_1 \ne j_2$, where $1 \le i_1, i_2 \le n_0$, $n_0 + 1 \le j_1, j_2 \le n_0 + n_1$, $\xi^{(i_1,i_2,j_1,j_2)} = E\big[I(y_{i_1} < y_{j_1}) - f_1\big]E\big[I(y_{i_2} < y_{j_2}) - f_1\big]$;

when $i_1 = i_2 = i,\ j_1 \ne j_2$,

$$\begin{aligned}
\xi^{(i_1,i_2,j_1,j_2)} &= E\Big\{E\big\{[I(y_{i_1} < y_{j_1}) - f_1][I(y_{i_2} < y_{j_2}) - f_1]|y_i\big\}\Big\} \\
&= E\Big\{E[I(y_i < y_{j_1}) - f_1]E[I(y_i < y_{j_2}) - f_1]\Big\} \\
&= E\Big[1 - F_1(Y_0) - f_1\Big]^2;
\end{aligned}$$

when $i_1 \neq i_2,\ j_1 = j_2 = j$,

$$\begin{aligned}
\xi^{(i_1,i_2,j_1,j_2)} &= E\Big\{E\big\{[I(y_{i_1} < y_{j_1}) - f_1][I(y_{i_2} < y_{j_2}) - f_1]|y_j\big\}\Big\} \\
&= E\Big\{E[I(y_{i_1} < y_j) - f_1]E[I(y_{i_2} < y_j) - f_1]\Big\} \\
&= E\Big[F_0(Y_1) - f_1\Big]^2;
\end{aligned}$$

and when $i_1 = i_2 = i,\ j_1 = j_2 = j$,

$$\begin{aligned}
\xi^{(i_1,i_2,j_1,j_2)} &= E\big[I(y_i < y_j) - f_1\big]\big[I(y_i < y_j) - f_1\big] \\
&= E\big[I(y_i < y_j)\big] - 2f_1E\big[I(y_i < y_j)\big] + f_1^2 \\
&= f_1 - f_1^2.
\end{aligned}$$

Thus

$$\begin{aligned}
\text{Var}(\hat{f}_1) &= \tfrac{1}{n_0n_1}\Big\{(n_1-1)E\Big[1 - F_1(Y_0) - f_1\Big]^2 + (n_0-1)E\Big[F_0(Y_1) - f_1\Big]^2 + (f_1 - f_1^2)\Big\} \\
&= \tfrac{n_1-1}{n_0n_1}E\Big[1 - F_1(Y_0) - f_1\Big]^2 + \tfrac{n_0-1}{n_0n_1}E\Big[F_0(Y_1) - f_1\Big]^2 + \tfrac{1}{n_0n_1}(f_1 - f_1^2)
\end{aligned}$$

So the emprical estimate of $\text{Var}(\hat{f}_1)$ under H_0 is

$$\hat{\sigma}_1^2 = \frac{n_1-1}{n_0^2n_1}\sum_{i=1}^{n_0}\left[\frac{1}{n_1}\sum_{j=n_0+1}^{n_0+n_1} I(y_i < y_j) - 1/2\right]^2 + \frac{n_0-1}{n_0n_1^2}\sum_{j=n_0+1}^{n_0+n_1}\left[\frac{1}{n_0}\sum_{i=1}^{n_0} I(y_i < y_j) - 1/2\right]^2 + \frac{1}{4n_0n_1}.$$

Similarly, we can derive the empirical estimate of $\text{Var}(\hat{f}_2)$.

1.4. Estimates of the covariances for Z_R, Z_A and Z_D under H_0

Using the notations in Table 5, the covariance of Z_R and Z_A under H_0 is

$$\gamma_{RA} = \widehat{\text{cov}}(Z_R, Z_A) = \frac{w_1\widehat{E}_{H_0}\Big(\big(\hat{f}_{2R} - 1/2\big)\big(\hat{f}_1 - 1/2\big)\Big)}{\hat{\sigma}_{2R}\hat{\sigma}_{1A}} + \frac{w_2\widehat{E}_{H_0}\Big(\big(\hat{f}_{2R} - 1/2\big)\big(\hat{f}_{12} - 1/2\big)\Big)}{\hat{\sigma}_{2R}\hat{\sigma}_{1A}},$$

where

$$\begin{aligned}
&\widehat{E}_{H_0}\Big(\big(\hat{f}_{2R} - 1/2\big)\big(\hat{f}_1 - 1/2\big)\Big) \\
&\quad = \frac{1}{n_0(n_0+n_1)}\sum_{i=1}^{n_0}\left[\frac{1}{n_1}\sum_{j=n_0+1}^{n_0+n_1}\big(I\{y_i < y_j\} - 1/2\big)\right]\left[\frac{1}{n_2}\sum_{k=n_0+n_1+1}^{n}\big(I\{y_i < y_k\} - 1/2\big)\right] \\
&\quad\quad + \frac{1}{n_1(n_0+n_1)}\sum_{j=n_0+1}^{n_0+n_1}\left[\frac{1}{n_0}\sum_{i=1}^{n_0}\big(I\{y_i < y_j\} - 1/2\big)\right]\left[\frac{1}{n_2}\sum_{k=n_0+n_1+1}^{n}\big(I\{y_j < y_k\} - 1/2\big)\right], \\
&\widehat{E}_{H_0}\Big(\big(\hat{f}_{2R} - 1/2\big)\big(\hat{f}_{12} - 1/2\big)\Big)
\end{aligned}$$

$$= \frac{n_0}{(n_0+n_1)n_2^2} \sum_{k=n_0+n_1+1}^{n} \left[\frac{1}{n_0} \sum_{i=1}^{n_0} \big(I\{y_i < y_k\} - 1/2 \big) \right] \left[\frac{1}{n_1} \sum_{j=n_0+1}^{n_0+n_1} \big(I\{y_j < y_k\} - 1/2 \big) \right] + \frac{n_1}{n_0+n_1} \widetilde{\sigma}_{12}^2,$$

and

$$\begin{aligned} \widetilde{\sigma}_{12}^2 &= \frac{n_2-1}{n_1^2 n_2} \sum_{j=n_0+1}^{n_0+n_1} \left[\frac{1}{n_2} \sum_{k=n_0+n_1+1}^{n} I(y_j < y_k) - \frac{1}{2} \right]^2 \\ &\quad + \frac{n_1-1}{n_1 n_2^2} \sum_{k=n_0+n_1+1}^{n} \left[\frac{1}{n_1} \sum_{j=n_0+1}^{n_0+n_1} I(y_j < y_k) - \frac{1}{2} \right]^2 + \frac{1}{4n_1 n_2}. \end{aligned}$$

The covariance of Z_A and Z_D under H_0 is

$$\gamma_{AD} = \widehat{\text{cov}}(Z_A, Z_D) = \frac{w_1 \widehat{E}_{H_0} \Big(\big(\hat{f}_1 - 1/2\big)\big(\hat{f}_{1D} - 1/2\big) \Big)}{\hat{\sigma}_{1A}\hat{\sigma}_{1D}} + \frac{w_2 \widehat{E}_{H_0} \Big(\big(\hat{f}_{12} - 1/2\big)\big(\hat{f}_{1D} - 1/2\big) \Big)}{\hat{\sigma}_{1A}\hat{\sigma}_{1D}},$$

where

$$\begin{aligned} &\widehat{E}_{H_0} \Big((\hat{f}_1 - 1/2)(\hat{f}_{1D} - 1/2) \Big) \\ &\quad = \frac{n_2}{(n_1+n_2)n_0^2} \sum_{i=1}^{n_0} \left[\frac{1}{n_1} \sum_{j=n_0+1}^{n_0+n_1} \big(I\{y_i < y_j\} - 1/2 \big) \right] \left[\frac{1}{n_2} \sum_{k=n_0+n_1+1}^{n} \big(I\{y_i < y_k\} - 1/2 \big) \right] + \\ &\frac{n_1}{n_1+n_2} \widetilde{\sigma}_1^2, \end{aligned}$$

$$\widetilde{\sigma}_1^2 = \frac{n_1-1}{n_0^2 n_1} \sum_{i=1}^{n_0} \left[\frac{1}{n_1} \sum_{j=n_0+1}^{n_0+n_1} I(y_i < y_j) - \frac{1}{2} \right]^2 + \frac{n_0-1}{n_0 n_1^2} \sum_{j=n_0+1}^{n_0+n_1} \left[\frac{1}{n_0} \sum_{i=1}^{n_0} I(y_i < y_j) - \frac{1}{2} \right]^2 + \frac{1}{4n_0 n_1}$$

and

$$\begin{aligned} &\widehat{E}_{H_0} \Big(\big(\hat{f}_{12} - 1/2\big)\big(\hat{f}_{1D} - 1/2\big) \Big) \\ &\quad = \frac{1}{n_1(n_1+n_2)} \sum_{j=n_0+1}^{n_0+n_1} \left[\frac{1}{n_0} \sum_{i=1}^{n_0} \big(I\{y_i < y_j\} - 1/2 \big) \right] \left[\frac{1}{n_2} \sum_{k=n_0+n_1+1}^{n} \big(I\{y_j < y_k\} - 1/2 \big) \right] \\ &\qquad + \frac{1}{n_2(n_1+n_2)} \sum_{k=n_0+n_1+1}^{n} \left[\frac{1}{n_0} \sum_{i=1}^{n_0} \big(I\{y_i < y_k\} - 1/2 \big) \right] \left[\frac{1}{n_1} \sum_{j=n_0+1}^{n_0+n_1} \big(I\{y_j < y_k\} - 1/2 \big) \right]. \end{aligned}$$

The covariance of Z_R and Z_D under H_0 is

$$\gamma_{RD} = \widehat{\text{cov}}(Z_R, Z_D) = \frac{\widehat{E}_{H_0} \Big(\big(\hat{f}_{2R} - 1/2\big)\big(\hat{f}_{1D} - 1/2\big) \Big)}{\hat{\sigma}_{2R}\hat{\sigma}_{1D}},$$

where

$$\widehat{E}_{H_0} \Big((\hat{f}_{2R} - 1/2)(\hat{f}_{1D} - 1/2) \Big)$$

$$
\begin{aligned}
&= \frac{n_1}{n_0(n_0+n_1)(n_1+n_2)} \sum_{i=1}^{n_0} \left[\frac{1}{n_1} \sum_{j=n_0+1}^{n_0+n_1} \big(I\{y_j < y_k\} - 1/2 \big) \right] \left[\frac{1}{n_2} \sum_{k=n_0+n_1+1}^{n} \big(I\{y_i < y_k\} - 1/2 \big) \right] \\
&\quad + \frac{1}{(n_0+n_1)(n_1+n_2)} \sum_{j=n_0+1}^{n_0+n_1} \left[\frac{1}{n_0} \sum_{i=1}^{n_0} \big(I\{y_i < y_j\} - 1/2 \big) \right] \left[\frac{1}{n_2} \sum_{k=n_0+n_1+1}^{n} \big(I\{y_j < y_k\} - 1/2 \big) \right] \\
&\quad + \frac{n_1}{n_2(n_0+n_1)(n_1+n_2)} \sum_{k=n_0+n_1+1}^{n} \left[\frac{1}{n_0} \sum_{i=1}^{n_0} \big(I\{y_i < y_k\} - 1/2 \big) \right] \left[\frac{1}{n_1} \sum_{j=n_0+1}^{n_0+n_1} \big(I\{y_j < y_k\} - 1/2 \big) \right] \\
&\quad + \frac{n_0 n_2 \widetilde{\sigma}_2^2}{(n_0+n_1)(n_1+n_2)},
\end{aligned}
$$

and

$$
\widetilde{\sigma}_2^2 = \frac{n_2-1}{n_0^2 n_2} \sum_{i=1}^{n_0} \left[\frac{1}{n_2} \sum_{k=n_0+n_1+1}^{n_0+n_1+n_2} I(y_i < y_k) - \frac{1}{2} \right]^2 + \frac{n_0-1}{n_0 n_2^2} \sum_{k=n_0+n_1+1}^{n_0+n_1+n_2} \left[\frac{1}{n_0} \sum_{i=1}^{n_0} I(y_i < y_k) - \frac{1}{2} \right]^2 + \frac{1}{4 n_0 n_2}.
$$

2. Additional simulation results without considering the covariates.

Tables

1) Tables S1 and S2 show the empirical results of the point and confidence interval estimation of λ_1 and λ_2 for $\beta_1 = 0.25$ and $\beta_1 = 0.5$, respectively.

2) Tables S3 and S4 show the empirical results of the point and confidence interval estimation of λ_{2R} for $\beta_1 = 0.25$ and $\beta_1 = 0.5$, respectively, under the recessive model.

3) Tables S5 and S6 show the empirical results of the point and confidence interval estimation of λ_{1A} for $\beta_1 = 0.25$ and $\beta_1 = 0.5$, respectively, under the additive model.

4) Tables S7 and S8 show the empirical results of the point and confidence interval estimation of λ_{1D} for $\beta_1 = 0.25$ and $\beta_1 = 0.5$, respectively, under the dominant model.

Results

In Tables S1 to S8, we report the empirical biases, the square root of mean square errors (sMSEs), the cover probabilities (CPs) and the interval lengthes (ILs) of the Standard Interval, the Wilson Interval and the Log-Delta Interval. From these tables, we find that the sMSE is decreasing with the increasing of the minor allele frequency (MAF) overall. The three interval estimation methods behave well with their empirical CPs being close to the nominal level of 95% when the MAFs are large. For instance, when $\beta_1 = 0.25$ and MAF = 0.25, the coverage probabilities of the Standard Interval, the Wilson Interval and the Log-Delta Interval for λ_2 are $94.6\%, 94.7\%$ and 94.7%, respectively. When the MAF is small, for example, MAF = 0.05, the performances of the above three procedures are optimistic to infer λ_2. Their empirical CPs are less than 95%. For example, when $\beta_1 = 0.25$ and MAF = 0.05, the empirical CPs of the Standard Interval, the Wilson Interval and the Log-Delta Interval for λ_2 are, respectively, 0.824, 0.844 and 0.848. This is because the number of subjects with genotype 2 is very small (the mean number of subjects with genotype 2 is 3.75 for $n = 1,500$), the asymptotic statistical property does not hold. We also find that the Wilson Interval and the Log-Delta Interval have almost the same performances. Overall, both the Log-Delta and Wilson Intervals work better than Standard Interval. In general, the Wilson Interval and the Log-Delta Interval have

larger CP and shorter IL than the Standard Interval.

Table S1. The empirical biases, sMSE, CP sand IL for $\beta_1 = 0.25$.

MAF	Bias	sMSE	CP	IL	CP	IL	CP	IL
			Standard Interval		Wilson Interval		Log-Delta Interval	
			For inferring $\lambda_1(= 1.43)$					
0.10	0.0465	0.113	0.927	0.408	0.933	0.406	0.933	0.406
0.15	-0.0085	0.092	0.953	0.369	0.951	0.367	0.951	0.367
0.20	-0.0122	0.092	0.937	0.353	0.938	0.351	0.938	0.352
0.25	-0.0016	0.087	0.948	0.348	0.950	0.347	0.950	0.348
0.30	0.0384	0.096	0.936	0.354	0.939	0.353	0.939	0.353
0.35	0.0088	0.091	0.953	0.364	0.954	0.363	0.955	0.363
0.40	-0.0232	0.098	0.944	0.382	0.944	0.380	0.944	0.380
0.45	-0.0052	0.101	0.948	0.404	0.947	0.402	0.948	0.402
0.50	-0.0030	0.109	0.960	0.435	0.960	0.433	0.961	0.433
			For inferring $\lambda_2(= 1.97)$					
0.10	0.0651	0.553	0.922	2.253	0.925	2.025	0.926	2.048
0.15	0.0160	0.350	0.932	1.382	0.932	1.329	0.932	1.336
0.20	0.0383	0.268	0.943	1.039	0.945	1.017	0.945	1.019
0.25	-0.0242	0.217	0.946	0.856	0.947	0.844	0.947	0.845
0.30	-0.0038	0.184	0.953	0.757	0.953	0.748	0.953	0.749
0.35	0.0373	0.176	0.953	0.704	0.954	0.697	0.954	0.698
0.40	0.0321	0.174	0.948	0.681	0.950	0.674	0.950	0.675
0.45	-0.0200	0.168	0.951	0.677	0.949	0.671	0.949	0.672
0.50	-0.0032	0.176	0.951	0.700	0.950	0.694	0.950	0.695

Table S2. The empirical biases, sMSE, CP and IL for $\beta_1 = 0.5$.

			Standard Interval		Wilson Interval		Log-Delta Interval	
MAF	Bias	sMSE	CP	IL	CP	IL	CP	IL
			For inferring $\lambda_1 (= 1.97)$					
0.10	0.0174	0.141	0.946	0.547	0.946	0.543	0.947	0.544
0.15	-0.0047	0.126	0.953	0.504	0.952	0.501	0.952	0.501
0.20	-0.0212	0.127	0.944	0.491	0.944	0.488	0.944	0.489
0.25	-0.0025	0.124	0.945	0.494	0.945	0.492	0.945	0.492
0.30	0.0072	0.125	0.956	0.506	0.955	0.504	0.955	0.504
0.35	0.0141	0.132	0.950	0.528	0.955	0.525	0.955	0.525
0.40	0.0010	0.143	0.951	0.558	0.950	0.555	0.950	0.555
0.45	-0.0010	0.154	0.945	0.595	0.944	0.591	0.944	0.591
0.50	-0.0180	0.164	0.949	0.647	0.950	0.642	0.950	0.643
			For inferring $\lambda_2 (= 3.58)$					
0.10	0.1701	0.978	0.910	3.688	0.921	3.353	0.924	3.387
0.15	0.0596	0.589	0.940	2.314	0.942	2.204	0.943	2.212
0.20	0.0011	0.447	0.947	1.779	0.945	1.728	0.945	1.732
0.25	0.0960	0.388	0.936	1.515	0.947	1.483	0.948	1.486
0.30	0.0085	0.350	0.946	1.394	0.949	1.369	0.949	1.371
0.35	0.0876	0.346	0.943	1.342	0.951	1.320	0.951	1.322
0.40	0.0698	0.345	0.940	1.346	0.946	1.324	0.946	1.326
0.45	0.0982	0.371	0.932	1.379	0.938	1.355	0.938	1.357
0.50	0.0489	0.372	0.949	1.467	0.952	1.439	0.952	1.441

Table S3. The empirical biases, sMSEs, CP and IL for $\beta_1 = \ln 1.2$ under the recessive model.

MAF	Bias	sMSE	CP	IL	CP	IL	CP	IL
			Standard Interval		Wilson Interval		Log-Delta Interval	
			For inferring $\lambda_2 (= 1.97)$					
0.10	0.0705	0.555	0.919	2.302	0.925	2.033	0.926	2.058
0.15	0.0438	0.342	0.942	1.363	0.948	1.313	0.949	1.319
0.20	0.0030	0.259	0.941	1.007	0.946	0.987	0.946	0.989
0.25	0.0016	0.206	0.955	0.817	0.949	0.806	0.950	0.808
0.30	0.0092	0.174	0.948	0.701	0.948	0.694	0.948	0.695
0.35	0.0752	0.172	0.922	0.620	0.930	0.615	0.930	0.616
0.40	-0.010	0.144	0.947	0.566	0.946	0.563	0.947	0.563
0.45	-0.020	0.135	0.948	0.528	0.945	0.525	0.945	0.525
0.50	-0.005	0.129	0.945	0.503	0.943	0.501	0.943	0.501

Table S4. The empirical biases, sMSE, CP and IL for $\beta_1 = \ln 1.4$ under the recessive model.

MAF	Bias	sMSE	CP	IL	CP	IL	CP	IL
			Standard Interval		Wilson Interval		Log-Delta Interval	
			For inferring $\lambda_2 (= 3.58)$					
0.10	0.2629	0.971	0.915	3.898	0.924	3.351	0.925	3.383
0.15	0.0535	0.593	0.933	2.264	0.930	2.160	0.931	2.168
0.20	0.0326	0.423	0.951	1.701	0.949	1.657	0.949	1.661
0.25	0.0544	0.367	0.927	1.401	0.930	1.376	0.931	1.378
0.30	0.0458	0.314	0.946	1.225	0.946	1.208	0.946	1.210
0.35	0.0121	0.281	0.951	1.109	0.952	1.096	0.952	1.097
0.40	-0.0157	0.270	0.942	1.036	0.941	1.025	0.942	1.026
0.45	0.0088	0.252	0.949	0.989	0.950	0.980	0.950	0.981
0.50	0.0028	0.238	0.963	0.959	0.962	0.950	0.962	0.951

Table S5. The empirical biases, sMSE, CP and IL for $\beta_1 = 0.25$ under the additive model.

MAF	Bias	sMSE	CP	IL	CP	IL	CP	IL
			Standard Interval		Wilson Interval		Log-Delta Interval	
			For inferring $\lambda_1(= 1.43)$					
0.10	0.0049	0.092	0.947	0.357	0.947	0.356	0.947	0.356
0.15	-0.0153	0.080	0.938	0.306	0.938	0.305	0.938	0.305
0.20	0.0082	0.072	0.945	0.278	0.945	0.277	0.945	0.277
0.25	-0.0013	0.065	0.957	0.260	0.957	0.260	0.957	0.260
0.30	-0.0029	0.062	0.954	0.249	0.954	0.249	0.954	0.249
0.35	0.0144	0.065	0.940	0.243	0.940	0.243	0.940	0.243
0.40	-0.0012	0.060	0.954	0.239	0.953	0.239	0.953	0.239
0.45	-0.0018	0.061	0.939	0.239	0.939	0.239	0.939	0.239
0.50	-0.0084	0.061	0.945	0.241	0.943	0.240	0.943	0.240
			For inferring $\lambda_2(= 1.97)$					
0.10	0.1365	0.590	0.913	2.370	0.918	2.056	0.920	2.081
0.15	0.0156	0.343	0.941	1.389	0.943	1.336	0.945	1.342
0.20	0.0327	0.270	0.933	1.038	0.938	1.016	0.938	1.019
0.25	0.0203	0.218	0.944	0.862	0.950	0.850	0.951	0.851
0.30	0.0090	0.187	0.950	0.757	0.954	0.748	0.954	0.750
0.35	0.0184	0.183	0.944	0.706	0.943	0.699	0.944	0.700
0.40	0.0032	0.169	0.953	0.678	0.951	0.672	0.951	0.673
0.45	0.0306	0.176	0.946	0.682	0.950	0.676	0.950	0.677
0.50	0.0324	0.178	0.949	0.699	0.950	0.692	0.950	0.693

Table S6. The empirical biases, sMSE, CP and IL for $\beta_1 = 0.5$ under the additive model.

MAF	Bias	sMSE	CP	IL	CP	IL	CP	IL
			Standard Interval		Wilson Interval		Log-Delta Interval	
			For inferring $\lambda_1(= 1.97)$					
0.10	0.0309	0.127	0.940	0.473	0.941	0.471	0.941	0.471
0.15	0.0077	0.104	0.943	0.413	0.945	0.412	0.945	0.412
0.20	0.0250	0.101	0.940	0.381	0.943	0.380	0.943	0.380
0.25	0.0185	0.095	0.950	0.362	0.948	0.361	0.948	0.361
0.30	0.0055	0.089	0.946	0.352	0.945	0.351	0.945	0.351
0.35	0.0415	0.099	0.921	0.346	0.925	0.345	0.925	0.345
0.40	-0.0017	0.088	0.952	0.345	0.952	0.344	0.952	0.344
0.45	-0.0060	0.089	0.949	0.348	0.949	0.347	0.949	0.347
0.50	0.0237	0.094	0.939	0.353	0.942	0.352	0.942	0.352
			For inferring $\lambda_2(= 3.58)$					
0.10	0.1818	0.934	0.907	3.877	0.912	3.376	0.914	3.409
0.15	0.0790	0.579	0.932	2.311	0.941	2.201	0.943	2.210
0.20	0.0969	0.455	0.945	1.782	0.950	1.731	0.951	1.735
0.25	0.0229	0.381	0.945	1.516	0.945	1.484	0.945	1.487
0.30	0.0670	0.355	0.948	1.398	0.949	1.373	0.949	1.376
0.35	0.0237	0.345	0.942	1.341	0.942	1.319	0.942	1.320
0.40	0.0638	0.344	0.946	1.338	0.950	1.316	0.951	1.318
0.45	0.1122	0.374	0.931	1.387	0.936	1.363	0.937	1.365
0.50	0.0645	0.375	0.946	1.469	0.951	1.440	0.952	1.443

Table S7. The empirical biases, sMSE, CP and IL for $\beta_1 = 0.25$ under the dominant model.

MAF	Bias	sMSE	CP	IL	CP	IL	CP	IL
			Standard Interval		Wilson Interval		Log-Delta Interval	
			For inferring $\lambda_1(=\lambda_2 = 1.97)$					
0.10	0.0066	0.137	0.952	0.539	0.951	0.536	0.951	0.536
0.15	-0.0033	0.128	0.935	0.492	0.933	0.490	0.933	0.490
0.20	0.0246	0.123	0.944	0.477	0.947	0.475	0.947	0.475
0.25	0.0054	0.123	0.944	0.480	0.945	0.478	0.945	0.478
0.30	0.0041	0.123	0.952	0.491	0.953	0.489	0.953	0.489
0.35	-0.0195	0.135	0.940	0.512	0.940	0.510	0.940	0.510
0.40	0.0297	0.135	0.954	0.540	0.955	0.537	0.955	0.537
0.45	0.0028	0.145	0.951	0.578	0.948	0.574	0.948	0.574
0.50	0.0109	0.161	0.945	0.625	0.945	0.620	0.945	0.621

Table S8. The empirical biases, sMSE, CP and IL for $\beta_1 = 0.5$ under the dominant model.

MAF	Bias	sMSE	CP	IL	CP	IL	CP	IL
			Standard Interval		Wilson Interval		Log-Delta Interval	
			For inferring $\lambda_1(=\lambda_2 = 3.58)$					
0.10	0.0256	0.244	0.956	1.000	0.957	0.991	0.957	0.991
0.15	0.0440	0.243	0.943	0.952	0.947	0.944	0.947	0.945
0.20	-0.0276	0.240	0.948	0.952	0.946	0.944	0.946	0.945
0.25	-0.0042	0.248	0.948	0.979	0.946	0.970	0.946	0.971
0.30	0.0434	0.262	0.943	1.020	0.947	1.010	0.947	1.011
0.35	0.0223	0.269	0.952	1.083	0.952	1.071	0.952	1.072
0.40	0.0179	0.294	0.943	1.152	0.947	1.138	0.947	1.139
0.45	-0.0165	0.321	0.940	1.248	0.937	1.230	0.937	1.232
0.50	0.0489	0.343	0.952	1.373	0.952	1.349	0.952	1.351

3. Additional simulation results for sample size $n = 1000$ and $n = 500$.

Consider the linear model $Y = \beta_0 + G\beta_1 + \epsilon$, where $Y = (y_1, y_2, \cdots, y_n)^\tau$ denotes the trait value, $G = (g_1, g_2, \cdots, g_n)^\tau$ denotes the genotype value at a single nucleotide polymorphism (SNP) locus with $g_i \in \{0, 1, 2\}$ being the count of a certain allele, and $\epsilon = (\epsilon_1, \epsilon_2, \cdots, \epsilon_n)^\tau$ denotes the random error with ϵ_i *i.i.d.* following a truncated generalized extreme value distribution (a heavy-tailed distribution), tGEV$(0, 0, 1, 0)$ with the shape parameter 0, the location parameter 0, the scale parameter 1 and the truncated point 0. We consider $\beta_0 = 0.5$, $\beta_1 \in \{0, 0.5\}$ and four minor allele frequencies (MAF-s) with 0.05, 0.15, 0.30 and 0.45. In Table S9 and S10, we report the empirical type I error rates for the sample size $n = 1000$ and $n = 500$, respectively. They shows that the proposed tests can control the type I error rates. KLH is too optimistic. Figure S1 shows the empirical power results of the Kruskal-Wallis test (KW-R, KW-A, KW-D), F test (F-R, F-A, F-D), the Jonckheere-Terpstra test (JT-R, JT-A,JT-D), and the proposed nonparametric test (Z_R, Z_A, Z_D). It indicates that the proposed test (Z_R, Z_A, Z_D) is more powerful than the other existing tests under the specific genetic model. The power results of KW-A, F-A, Z_A and MAX3 are shown in Figure S2. Under the additive and dominant models with MAF being large, MAX3 is more powerful than KW-A, F-A and Z_A. Under the additive model, the Z_A performs the best among them since the data are generated under it. Overall MAX3 is the most robust among them.

Table S9. The empirical type I error rates of the Kruskal-Wallis test (KW-R, KW-A, KW-D), the F test (F-R, F-A, F-D), the Jonckheere-Terpstra test (JT-R, JT-A, JT-D), the KLH and the proposed test (Z_R, Z_A, Z_D) and the proposed MAX3. The nominal level is 0.05 and 2,000 replicates are conducted. (the sample size is 1000).

MAF	KW-R	KW-A	KW-D	F-R	F-A	F-D	JT-R	JT-A	JT-D	KLH	Z_R	Z_A	Z_D	MAX3
0.05	0.040	0.041	0.048	0.050	0.051	0.047	0.037	0.041	0.044	0.213	0.007	0.049	0.048	0.024
0.15	0.051	0.047	0.053	0.043	0.050	0.046	0.045	0.035	0.043	0.060	0.046	0.046	0.050	0.044
0.30	0.053	0.055	0.045	0.051	0.054	0.052	0.040	0.041	0.033	0.051	0.050	0.049	0.042	0.049
0.45	0.043	0.049	0.058	0.049	0.058	0.059	0.041	0.046	0.048	0.056	0.042	0.056	0.056	0.052

Table S10. The empirical type I error rates of the Kruskal-Wallis test (KW-R, KW-A, KW-D), the F test (F-R, F-A, F-D), the Jonckheere-Terpstra test (JT-R, JT-A, JT-D), the KLH and the proposed test (Z_R, Z_A, Z_D) and the proposed MAX3. The nominal level is 0.05 and 2,000 replicates are conducted. (the sample size is 500).

MAF	KW-R	KW-A	KW-D	F-R	F-A	F-D	JT-R	JT-A	JT-D	KLH	Z_R	Z_A	Z_D	MAX3
0.05	0.029	0.020	0.036	0.031	0.029	0.029	0.026	0.029	0.030	0.196	5e-04	0.037	0.035	0.018
0.15	0.049	0.048	0.048	0.047	0.049	0.047	0.042	0.040	0.031	0.085	0.041	0.043	0.046	0.037
0.30	0.048	0.044	0.052	0.049	0.051	0.050	0.039	0.040	0.039	0.054	0.048	0.042	0.047	0.040
0.45	0.057	0.058	0.043	0.053	0.050	0.047	0.042	0.037	0.034	0.051	0.052	0.042	0.042	0.046

Figure S1. The empirical powers of the Kruskal-Wallis test, the F test, the Jonckheere-Terpstra test and the proposed test when $\beta_1 = 0.5$. The first column is for the sample size $n = 1000$ and the second column is for $n = 500$.

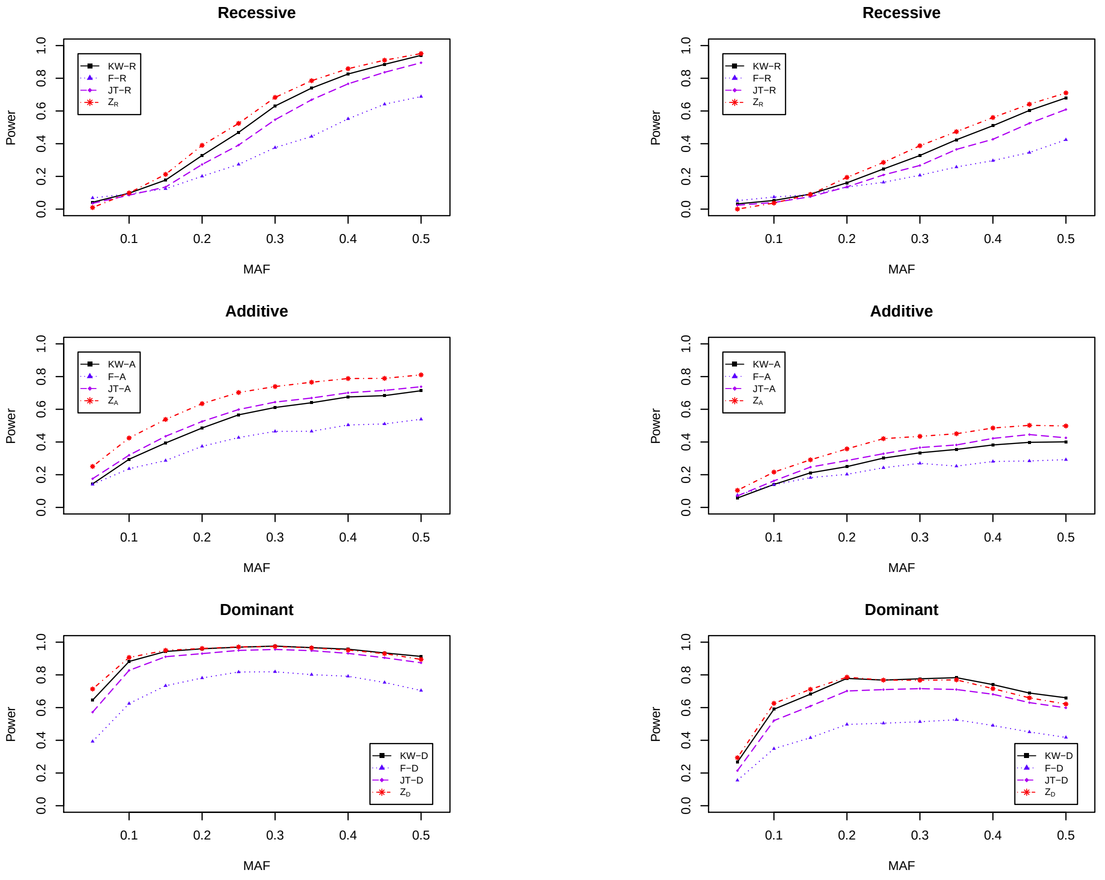

Figure S2. The empirical powers of KW-A, F-A, Z_A and MAX3 when $\beta_1 = 0.5$. The first column is for the sample size $n = 1000$ and the second column is for $n = 500$.

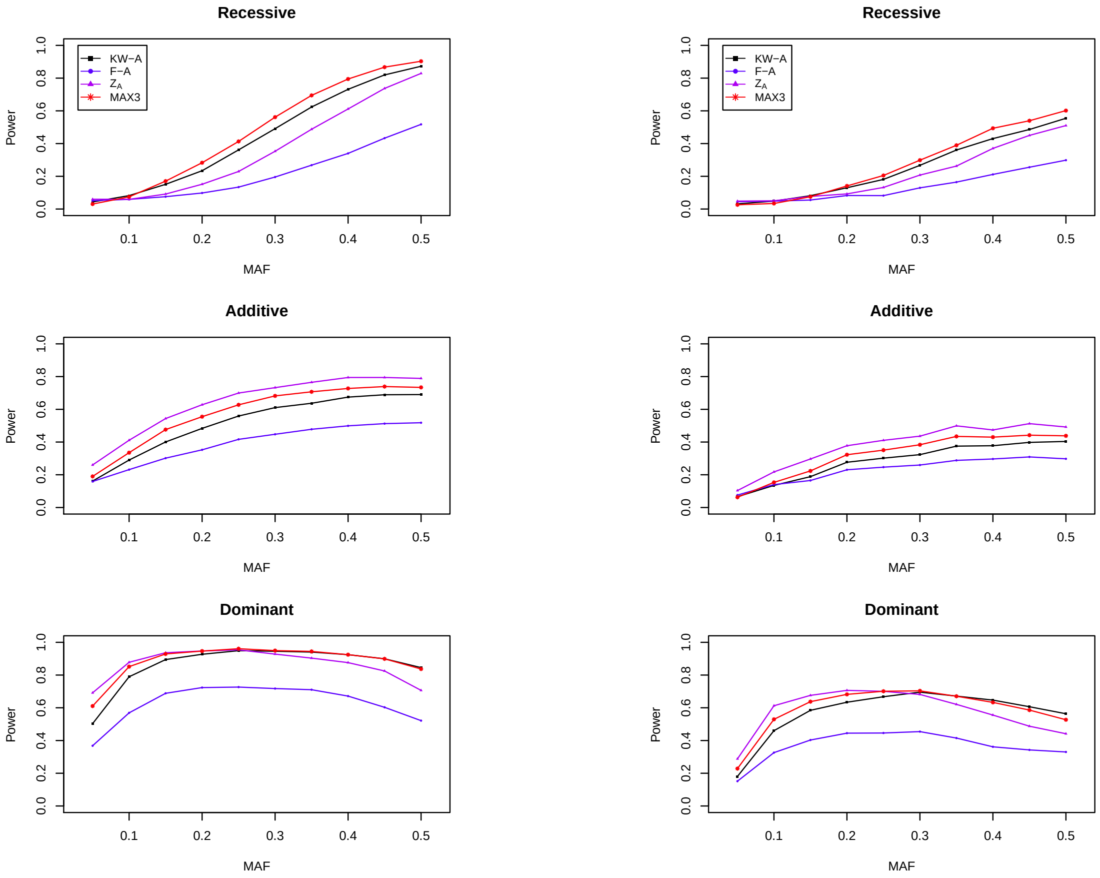

4. Estimation procedures considering the covariates.

Adjusting for the effects of covariates is commonly come across in population-based genetic association studies. For example, the potential population structure, a very important confounder factor usually leads to many false positive findings. So adjusting for population stratification has become a routine when doing the association analysis. Suppose that X is $n \times p$ matrix for a p-dimensional covariate. Using the notations in the main text, we first make the orthogonal projection of y $\left(\widehat{=}(y_1, y_2, \cdots, y_n)^\tau\right)$ on X. Denote the residual by $\tilde{\epsilon}$ $\left(\widehat{=}(\tilde{\epsilon}_1, \tilde{\epsilon}_2, \cdots, \tilde{\epsilon}_n)^\tau\right)$, where $\tilde{\epsilon} = y - X(X^\tau X)^{-1}X^\tau y$. Then we substitute y_i by $\tilde{\epsilon}_i$ and conduct the point and confidence interval estimation procedures as in the main text.

5. Simulation studies considering the covariates.

Simulation designs

Consider the linear model $Y = \beta_0 + X\gamma + G\beta_1 + \epsilon$, where X follow the standard normal $N(0, 1)$. Let $\beta_0 = 0.5$ and $\beta_1 \in \{0.25, 0.5\}$. 10 MAFs with 0.05, 0.10, 0.15, 0.20, 0.25, 0.30, 0.35, 0.40, 0.45, 0.50 are considered. The sample size is fixed to be 1,500 and 2,000 replicates are conducted to calculate the empirical biases, sMSEs, CPs and ILs for each scenario.

Tables

1) Tables S11 and S12 shows the empirical results of the point and confidence interval estimation of λ_1 and λ_2 for $\beta_1 = 0.25$ and $\beta_1 = 0.5$, respectively.

2) Tables S13 and S14 shows the empirical results of the point and confidence interval estimation of λ_{2R} for $\beta_1 = 0.25$ and $\beta_1 = 0.5$, respectively under the recessive model.

3) Tables S15 and S16 shows the empirical results of the point and confidence interval estimation of λ_{1A} for $\beta_1 = 0.25$ and $\beta_1 = 0.5$, respectively, under the additive model.

4) Tables S17 and S18 shows the empirical results of the point and confidence interval estimation of λ_{1D} for $\beta_1 = 0.25$ and $\beta_1 = 0.5$, respectively, under the dominant model.

Results

Similar phenomena are observed to those without considering the covariates. The sMSE is decreasing with the increasing of the MAF overall. The three interval estimation methods behave well and keep the CPs correctly when the MAF is large. When the MAF is small, the performances of the above three confidence interval procedures are a little bit optimistic to infer λ_2 with the empirical CPs being less than 95%. The reason is that the number of subjects with genotype 2 is very small and the asymptotic statistical property does not hold. The Wilson Interval and the Log-Delta Interval have almost the same performances. Overall the Log-Delta and the Wilson method have larger CP and shorter IL than the Standard Interval.

Table S11. The empirical biases, sMSE, CP and IL for $\beta_1 = 0.25$.

MAF	Bias	sMSE	CP	IL	CP	IL	CP	IL
			Standard Interval		Wilson Interval		Log-Delta Interval	
			For inferring $\lambda_1(= 1.43)$					
0.10	-0.0130	0.107	0.936	0.407	0.936	0.406	0.936	0.406
0.15	-0.0109	0.093	0.948	0.368	0.948	0.367	0.948	0.367
0.20	0.0028	0.089	0.948	0.352	0.947	0.351	0.947	0.351
0.25	-0.0115	0.087	0.952	0.349	0.951	0.348	0.951	0.348
0.30	0.0181	0.092	0.942	0.354	0.942	0.353	0.942	0.353
0.35	0.0060	0.092	0.948	0.363	0.948	0.362	0.949	0.362
0.40	-0.0055	0.097	0.947	0.380	0.949	0.378	0.949	0.379
0.45	0.0165	0.104	0.948	0.405	0.947	0.403	0.947	0.403
0.50	0.0020	0.110	0.946	0.433	0.946	0.431	0.946	0.431
			For inferring $\lambda_2(= 1.97)$					
0.10	0.0583	0.568	0.914	2.242	0.918	2.018	0.920	2.042
0.15	0.0384	0.352	0.944	1.386	0.947	1.333	0.949	1.340
0.20	-0.0053	0.259	0.949	1.037	0.950	1.014	0.951	1.017
0.25	0.0216	0.211	0.957	0.857	0.956	0.844	0.958	0.846
0.30	0.0041	0.189	0.948	0.761	0.949	0.752	0.950	0.753
0.35	0.0341	0.177	0.952	0.705	0.955	0.698	0.955	0.699
0.40	-0.0025	0.170	0.949	0.676	0.952	0.670	0.952	0.671
0.45	0.0175	0.171	0.952	0.679	0.949	0.673	0.949	0.674
0.50	0.0251	0.178	0.949	0.697	0.952	0.690	0.952	0.691

Table S12. The empirical biases, sMSE, CP and IL for $\beta_1 = 0.5$.

MAF	Bias	sMSE	CP	IL	CP	IL	CP	IL
			Standard Interval		Wilson Interval		Log-Delta Interval	
			For inferring $\lambda_1(= 1.97)$					
0.10	0.0018	0.137	0.953	0.546	0.953	0.543	0.953	0.543
0.15	-0.0072	0.129	0.944	0.503	0.945	0.501	0.945	0.501
0.20	-0.0171	0.126	0.943	0.490	0.943	0.488	0.944	0.488
0.25	-0.0126	0.128	0.935	0.493	0.935	0.491	0.935	0.491
0.30	0.0021	0.130	0.948	0.504	0.950	0.502	0.950	0.502
0.35	0.0119	0.137	0.934	0.525	0.936	0.522	0.936	0.523
0.40	0.0289	0.141	0.954	0.556	0.955	0.552	0.955	0.553
0.45	-0.0004	0.149	0.950	0.593	0.951	0.589	0.951	0.590
0.50	0.0006	0.162	0.951	0.643	0.951	0.637	0.951	0.638
			For inferring $\lambda_2(= 3.58)$					
0.10	0.1578	0.918	0.924	3.789	0.932	3.329	0.933	3.361
0.15	0.1019	0.562	0.942	2.302	0.946	2.193	0.946	2.202
0.20	0.0341	0.455	0.939	1.774	0.942	1.723	0.942	1.727
0.25	0.0913	0.401	0.941	1.516	0.945	1.484	0.946	1.487
0.30	0.0377	0.356	0.945	1.388	0.949	1.363	0.949	1.365
0.35	0.0097	0.337	0.950	1.334	0.950	1.312	0.950	1.314
0.40	-0.0105	0.338	0.949	1.340	0.944	1.318	0.945	1.320
0.45	0.0229	0.346	0.947	1.376	0.953	1.352	0.954	1.354
0.50	0.0817	0.375	0.944	1.461	0.949	1.433	0.949	1.435

Table S13. The empirical biases, sMSE, CP and IL for $\beta_1 = 0.25$ under the recessive model.

MAF	Bias	sMSE	CP	IL	CP	IL	CP	IL
			Standard Interval		Wilson Interval		Log-Delta Interval	
			For inferring $\lambda_2(= 1.97)$					
0.10	0.0834	0.562	0.915	2.282	0.923	2.046	0.926	2.070
0.15	0.0470	0.352	0.941	1.381	0.942	1.328	0.943	1.334
0.20	0.0371	0.262	0.942	1.016	0.946	0.995	0.947	0.998
0.25	0.0136	0.216	0.939	0.822	0.938	0.811	0.939	0.812
0.30	-0.0067	0.179	0.943	0.699	0.941	0.693	0.942	0.693
0.35	0.0077	0.157	0.954	0.619	0.953	0.615	0.953	0.615
0.40	-0.0026	0.145	0.945	0.566	0.946	0.563	0.946	0.563
0.45	0.0328	0.138	0.938	0.526	0.939	0.523	0.939	0.524
0.50	0.0156	0.126	0.956	0.502	0.956	0.500	0.956	0.500

Table S14. The empirical biases, sMSE, CP and IL for $\beta_1 = 0.5$ under the recessive model.

MAF	Bias	sMSE	CP	IL	CP	IL	CP	IL
			Standard Interval		Wilson Interval		Log-Delta Interval	
			For inferring $\lambda_2(= 3.58)$					
0.10	0.1637	0.917	0.924	3.678	0.934	3.300	0.935	3.332
0.15	0.0779	0.565	0.931	2.245	0.937	2.142	0.937	2.151
0.20	0.0565	0.430	0.933	1.696	0.937	1.651	0.937	1.655
0.25	0.0210	0.360	0.942	1.401	0.941	1.376	0.941	1.378
0.30	-0.0302	0.308	0.952	1.232	0.948	1.215	0.948	1.217
0.35	-0.0228	0.275	0.953	1.108	0.951	1.095	0.951	1.096
0.40	0.0027	0.265	0.948	1.034	0.946	1.023	0.946	1.024
0.45	0.0100	0.248	0.945	0.986	0.947	0.977	0.948	0.977
0.50	0.0298	0.241	0.954	0.960	0.955	0.951	0.955	0.952

Table S15. The empirical biases, sMSE, CP and IL for $\beta_1 = 0.25$ under the additive model.

MAF	Bias	sMSE	CP	IL	CP	IL	CP	IL
			Standard Interval		Wilson Interval		Log-Delta Interval	
			For inferring $\lambda_1(= 1.43)$					
0.10	-0.0013	0.093	0.935	0.357	0.939	0.356	0.939	0.356
0.15	0.0138	0.082	0.942	0.307	0.943	0.306	0.944	0.306
0.20	-0.0039	0.071	0.949	0.278	0.948	0.277	0.948	0.277
0.25	-0.0146	0.069	0.937	0.260	0.937	0.259	0.937	0.259
0.30	-0.0089	0.063	0.948	0.249	0.947	0.248	0.947	0.248
0.35	-0.0025	0.062	0.943	0.243	0.944	0.242	0.944	0.242
0.40	0.0122	0.063	0.937	0.240	0.938	0.239	0.939	0.239
0.45	-0.0058	0.061	0.949	0.238	0.948	0.238	0.948	0.238
0.50	0.0094	0.062	0.949	0.240	0.950	0.240	0.950	0.240
			For inferring $\lambda_2(= 1.97)$					
0.10	0.0944	0.602	0.925	2.330	0.925	2.063	0.927	2.089
0.15	0.0268	0.351	0.937	1.396	0.938	1.342	0.939	1.349
0.20	0.0214	0.258	0.948	1.040	0.947	1.017	0.948	1.020
0.25	0.0006	0.213	0.946	0.858	0.949	0.845	0.949	0.847
0.30	-0.0130	0.190	0.947	0.756	0.949	0.747	0.949	0.748
0.35	0.0181	0.176	0.947	0.703	0.950	0.696	0.950	0.696
0.40	0.0260	0.176	0.942	0.682	0.944	0.676	0.945	0.677
0.45	-0.0045	0.170	0.948	0.677	0.949	0.670	0.949	0.671
0.50	0.0165	0.179	0.944	0.699	0.947	0.692	0.947	0.693

Table S16. The empirical biases, sMSE, CP and IL for $\beta_1 = 0.5$ under the additive model.

MAF	Bias	sMSE	CP	IL	CP	IL	CP	IL
			Standard Interval		Wilson Interval		Log-Delta Interval	
			For inferring $\lambda_1(= 1.97)$					
0.10	-0.0420	0.129	0.929	0.474	0.926	0.472	0.926	0.472
0.15	0.0148	0.106	0.944	0.412	0.947	0.410	0.947	0.410
0.20	-0.0184	0.100	0.940	0.379	0.941	0.378	0.941	0.378
0.25	-0.0038	0.092	0.942	0.361	0.941	0.360	0.941	0.360
0.30	0.0370	0.094	0.937	0.350	0.939	0.349	0.940	0.349
0.35	0.0065	0.086	0.953	0.344	0.954	0.344	0.954	0.344
0.40	-0.0024	0.090	0.936	0.342	0.937	0.342	0.937	0.342
0.45	-0.0004	0.085	0.951	0.346	0.952	0.345	0.952	0.345
0.50	-0.0075	0.091	0.936	0.350	0.933	0.349	0.933	0.349
			For inferring $\lambda_2(= 3.58)$					
0.10	0.1061	0.942	0.924	3.797	0.925	3.330	0.927	3.362
0.15	0.0266	0.557	0.940	2.284	0.943	2.176	0.943	2.185
0.20	0.0542	0.454	0.940	1.768	0.941	1.718	0.943	1.722
0.25	0.0206	0.377	0.947	1.511	0.947	1.479	0.947	1.482
0.30	0.0519	0.348	0.953	1.391	0.959	1.366	0.959	1.368
0.35	-0.0056	0.326	0.955	1.333	0.954	1.311	0.954	1.313
0.40	0.0088	0.343	0.932	1.327	0.935	1.305	0.935	1.307
0.45	-0.0149	0.329	0.959	1.375	0.955	1.351	0.955	1.353
0.50	0.0161	0.364	0.949	1.445	0.943	1.417	0.944	1.419

Table S17. The empirical biases, sMSE, CP and IL for $\beta_1 = 0.25$ under the dominant model.

MAF	Bias	sMSE	CP	IL	CP	IL	CP	IL
			Standard Interval		Wilson Interval		Log-Delta Interval	
			For inferring $\lambda_1 (= \lambda_2 = 1.97)$					
0.10	0.0424	0.143	0.940	0.538	0.944	0.534	0.944	0.535
0.15	0.0049	0.124	0.953	0.491	0.953	0.489	0.953	0.489
0.20	-0.0106	0.123	0.947	0.478	0.947	0.476	0.948	0.476
0.25	-0.0307	0.126	0.947	0.479	0.943	0.477	0.943	0.477
0.30	-0.0145	0.125	0.944	0.490	0.944	0.487	0.944	0.488
0.35	0.0130	0.133	0.941	0.510	0.942	0.507	0.943	0.508
0.40	-0.0369	0.141	0.943	0.538	0.939	0.535	0.939	0.535
0.45	0.0020	0.146	0.949	0.573	0.950	0.569	0.950	0.570
0.50	0.0008	0.162	0.944	0.623	0.947	0.618	0.947	0.619

Table S18. The empirical biases, sMSE, CP and IL for $\beta_1 = 0.5$ under the dominant model.

MAF	Bias	sMSE	CP	IL	CP	IL	CP	IL
			Standard Interval		Wilson Interval		Log-Delta Interval	
			For inferring $\lambda_1 (= \lambda_2 = 3.58)$					
0.10	-0.0286	0.250	0.951	0.999	0.947	0.989	0.947	0.990
0.15	0.0438	0.248	0.943	0.953	0.944	0.945	0.944	0.945
0.20	0.0088	0.242	0.946	0.952	0.945	0.944	0.945	0.945
0.25	0.0437	0.247	0.948	0.974	0.952	0.966	0.952	0.966
0.30	0.0077	0.263	0.946	1.017	0.946	1.007	0.946	1.008
0.35	0.0115	0.273	0.951	1.071	0.949	1.060	0.949	1.061
0.40	-0.0132	0.291	0.943	1.148	0.941	1.134	0.941	1.135
0.45	-0.0010	0.310	0.952	1.248	0.951	1.230	0.951	1.231
0.50	0.0269	0.352	0.942	1.353	0.944	1.330	0.945	1.332

6. The empirical type I error rates under the nominal significance level of 0.0005.

We consider the same settings as Table 3 in the main text except for the nominal significance level. Here we compare the type I error rates of all considered procedures including KW (KW-R, KW-A, KW-D), F (F-R, F-A, F-D), JT (JT-R, JT-A, JT-D), KLH, and the proposed test (Z_R, Z_A,Z_D), and the proposed MAX3 under the nominal significance level of 0.0005. From Table S19, it can be seen that the proposed test NRT (Z_R, Z_A,Z_D) can also control the type I error under a relatively stringent nominal significance level.

Table S19. The empirical type I error rates of the Kruskal-Wallis test (KW-R, KW-A, KW-D), the F test (F-R, F-A, F-D), the Jonckheere-Terpstra test (JT-R, JT-A, JT-D), the KLH and the proposed test (Z_R, Z_A, Z_D) and the proposed MAX3. The nominal level is 5×10^{-4} and 100,000 replicates are conducted.

MAF	KW-R	KW-A	KW-D	F-R	F-A	F-D	JT-R	JT-A	JT-D	KLH	Z_R	Z_A	Z_D	MAX3
0.05	0.00003	0.00025	0.00049	0.00466	0.00060	0.00055	0	0	0	0.08314	0.00009	0.00047	0.00038	0.00024
0.15	0.00052	0.00055	0.00046	0.00113	0.00061	0.00056	0	0	0	0.00200	0.00028	0.00046	0.00044	0.00045
0.30	0.00038	0.00042	0.00056	0.00061	0.00055	0.00050	0	0	0	0.00077	0.00030	0.00047	0.00052	0.00045
0.45	0.00058	0.00061	0.00051	0.00056	0.00053	0.00065	0	0	0	0.00075	0.00046	0.00040	0.00045	0.00054

7. P-values of KW-A, F-A, Z_A, and MAX3 for 25 SNPs in the gene PTPN22 and 45 SNPs in the genomic region of 6p21.33.

Tables

1) Tables S20 shows the p-values of testing H_0 for 25 SNPs in the gene PTPN22 conferring risk to the anti-CCP using KW-A, F-A, Z_A, and MAX3. The *permu* is the permutation method to calculate the statistical significance of MAX3.

2) Tables S21 shows the p-values of testing H_0 for 45 SNPs in the genomic region of 6p21.33 conferring risk to the anti-CCP using KW-A, F-A, Z_A, and MAX3. The *permu* is the permutation method to calculate the statistical significance of MAX3.

Table S20. The p-values of testing H_0 for 25 SNPs in the gene PTPN22 conferring risk to the anti-CCP.

snpid	KW-A	F-A	Z_A	MAX3	*permu*
rs2040041	0.9589	0.9899	0.7888	0.9551	0.9570
rs12117799	0.9431	0.8266	0.9776	0.9672	0.9690
rs2797409	0.0794	0.7691	0.5018	0.0940	0.1120
rs3827733	0.5513	0.5788	0.6147	0.4586	0.4560
rs3811021	0.5070	0.9787	0.9310	0.4936	0.4930
rs2476599	0.9352	0.9079	0.7410	0.9327	0.9220
rs3789607	0.5746	0.5987	0.4623	0.5469	0.5420
rs2476601	0.3752	0.5997	0.7112	0.5788	0.6020
rs1217407	0.0847	0.7744	0.5115	0.1035	0.0940
rs1217418	0.1140	0.6415	0.3568	0.1336	0.1250
rs6665194	0.1883	0.7684	0.4779	0.2283	0.2130
rs12566340	0.0708	0.8846	0.6416	0.1072	0.1030
rs7529353	0.0787	0.8557	0.6000	0.1100	0.1150
rs2358994	0.0116	0.3808	0.3916	0.0182	0.0140
rs1217394	0.7263	0.5121	0.4809	0.6718	0.6760
rs1217392	0.0599	0.2070	0.1920	0.0511	0.0580
rs10745340	0.0573	0.2173	0.2021	0.0489	0.0430
rs1217401	0.2528	0.5313	0.6416	0.2811	0.3070
rs17464525	0.5969	0.3292	0.4362	0.5128	0.4920
rs971173	0.2691	0.4795	0.5837	0.2836	0.2720
rs1217390	0.7590	0.8411	0.9552	0.8750	0.8620
rs878129	0.4384	0.8673	0.7768	0.6844	0.6760
rs11811771	0.0169	0.6574	0.9772	0.1512	0.1230
rs11102703	0.8298	0.7405	0.6479	0.8075	0.8010
rs7545038	0.9610	0.5425	0.7980	0.9599	0.9630

Table S21. The p-values of testing H_0 for 45 SNPs in the genomic region of 6p21.33 conferring risk to the anti-CCP.

snpid	KW	F-A	Z_A	MAX3	*permu*
rs2523467	0.0856	0.1480	0.2162	0.1194	0.1120
rs6940467	0.0955	0.3216	0.4080	0.3161	0.3500
rs12660382	0.1171	0.1934	0.3016	0.2207	0.1910
rs2395488	0.4095	0.6721	0.4135	0.4116	0.4040
rs2248372	0.3697	0.5921	0.4456	0.3856	0.3860
rs2248373	0.5770	0.9490	0.9282	0.7502	0.7390
rs2248462	0.2231	0.3514	0.2578	0.2329	0.2040
rs2516513	0.1731	0.4460	0.3607	0.2588	0.2600
rs2516424	0.3807	0.6554	0.4134	0.3866	0.3780
rs2248617	0.2046	0.6800	0.4369	0.2905	0.2990
rs3828893	0.7798	0.8243	0.9176	0.8234	0.8870
rs3749946	0.9459	0.8279	0.7306	0.9290	0.9280
rs3099844	0.0072	0.0020	0.0029	0.0054	0.0010
rs2905722	0.9811	0.7983	0.8496	0.9798	0.9820
rs2523647	0.3071	0.0937	0.1757	0.3019	0.2940
rs2516509	0.3130	0.4269	0.3078	0.3160	0.3090
rs2523710	0.1558	0.0665	0.1903	0.1335	0.1430
rs2905747	0.8230	0.6261	0.7525	0.8029	0.8150
rs2516415	0.1799	0.8495	0.7320	0.4414	0.4130
rs3130922	0.5966	0.3907	0.3982	0.5451	0.5320
rs3828903	0.6728	0.5544	0.8029	0.6776	0.6580
rs3828914	0.7357	0.6613	0.9008	0.7627	0.7540

Table S21. *continued*

snpid	KW	F-A	Z_A	MAX3	*permu*
rs2855812	0.1722	0.4002	0.2765	0.2124	0.1980
rs3134899	0.8547	0.8923	0.8985	0.9263	0.9160
rs2844498	0.8359	0.9456	0.7382	0.8322	0.8240
rs2246618	0.0112	0.5488	0.2134	0.0635	0.0500
rs2516400	0.0323	0.5605	0.3749	0.1273	0.1000
rs2516399	0.4060	0.3258	0.2750	0.3828	0.4210
rs2516398	0.1151	0.5114	0.4601	0.2599	0.2690
rs2246986	0.7166	0.7110	0.5617	0.7516	0.8000
rs2844494	0.1350	0.4653	0.4804	0.2739	0.2690
rs9267444	0.0159	0.1336	0.2130	0.0168	0.0140
rs3093998	0.3870	0.5501	0.5752	0.4703	0.4790
rs3130637	0.3048	0.6483	0.6023	0.2510	0.2590
rs3132454	0.0221	0.0011	0.0074	0.0165	0.0180
rs3093993	0.3048	0.6483	0.6045	0.2506	0.2470
rs3095227	0.3275	0.5598	0.5635	0.2596	0.2600
rs2259435	0.3330	0.0535	0.1688	0.3126	0.3060
rs3093983	0.2557	0.3953	0.3426	0.1661	0.1720
rs3130055	0.2463	0.5310	0.5788	0.2525	0.2730
rs3093978	0.2584	0.4140	0.3603	0.1691	0.1580
rs2734583	0.0004	0.0009	0.0014	0.0008	0.0005
rs2071596	0.8856	0.5367	0.6715	0.8637	0.8520
rs2516393	0.2584	0.4140	0.3587	0.1688	0.1470
rs2844509	0.0977	0.0704	0.1050	0.0862	0.0940

8. Simulation results for the random error term following the normal distribution.

Consider the linear model $Y = \beta_0 + G\beta_1 + \epsilon$, where $Y = (y_1, y_2, \cdots, y_n)^\tau$ denotes the trait value, $G = (g_1, g_2, \cdots, g_n)^\tau$ denotes the genotype value at a single nucleotide polymorphism (SNP) locus with $g_i \in \{0, 1, 2\}$ being the count of a certain allele, and $\epsilon = (\epsilon_1, \epsilon_2, \cdots, \epsilon_n)^\tau$ denotes the random error with ϵ_i *i.i.d.* following a normal distribution with the mean μ and the standard deviation b. We consider $\beta_0 = 0.5$, $\beta_1 \in \{0, \ln 1.2, \ln 1.4\}$, MAF$\in \{0.05, 0.15, 0.30, 0.45\}$, and $\mu = 0, b = 4$. The nominal significant level is 0.05 and 2,000 replicates are conducted. In Table S22, we report the empirical type I error rates with the sample size $n = 1500$. It shows that our proposed test can control the type I error rate. Figure S3 shows the empirical power results of the Kruskal-Wallis test (KW-R, KW-A, KW-D), the F test (F-R, F-A, F-D), the Jonckheere-Terpstra test (JT-R, JT-A,JT-D), and the proposed test (Z_R, Z_A, Z_D). It indicates that the F test (F-R, F-A, F-D) is most powerful among them. This is reasonable because F test is constructed based on the assumption of normal error. The power results of KW-A, F-A, Z_A and MAX3 are shown in Figure S4.

Table S22. The empirical type I error rates of the Kruskal-Wallis test (KW-R, KW-A, KW-D), the F test (F-R, F-A, F-D), the Jonckheere-Terpstra test (JT-R, JT-A, JT-D), the KLH and the proposed test (Z_R, Z_A, Z_D) and the proposed MAX3 when the random error term follows the normal distribution.

MAF	KW-R	KW-A	KW-D	F-R	F-A	F-D	JT-R	JT-A	JT-D	KLH	Z_R	Z_A	Z_D	MAX3
0.05	0.043	0.044	0.044	0.046	0.047	0.047	0.038	0.042	0.034	0.169	0.017	0.046	0.043	0.030
0.15	0.048	0.051	0.050	0.053	0.051	0.052	0.041	0.039	0.039	0.058	0.048	0.051	0.050	0.047
0.30	0.044	0.049	0.053	0.042	0.054	0.052	0.035	0.043	0.043	0.054	0.042	0.051	0.053	0.050
0.45	0.050	0.040	0.047	0.048	0.050	0.042	0.041	0.036	0.036	0.043	0.049	0.048	0.046	0.042

Figure S3. **The empirical powers of the Kruskal-Wallis test, the F test, the Jonckheere-Terpstra test and the proposed tests when the random error term follows the normal distribution. The first column is for $\beta_1 = \ln 1.2$ and the second column is for $\beta_1 = \ln 1.4$.**

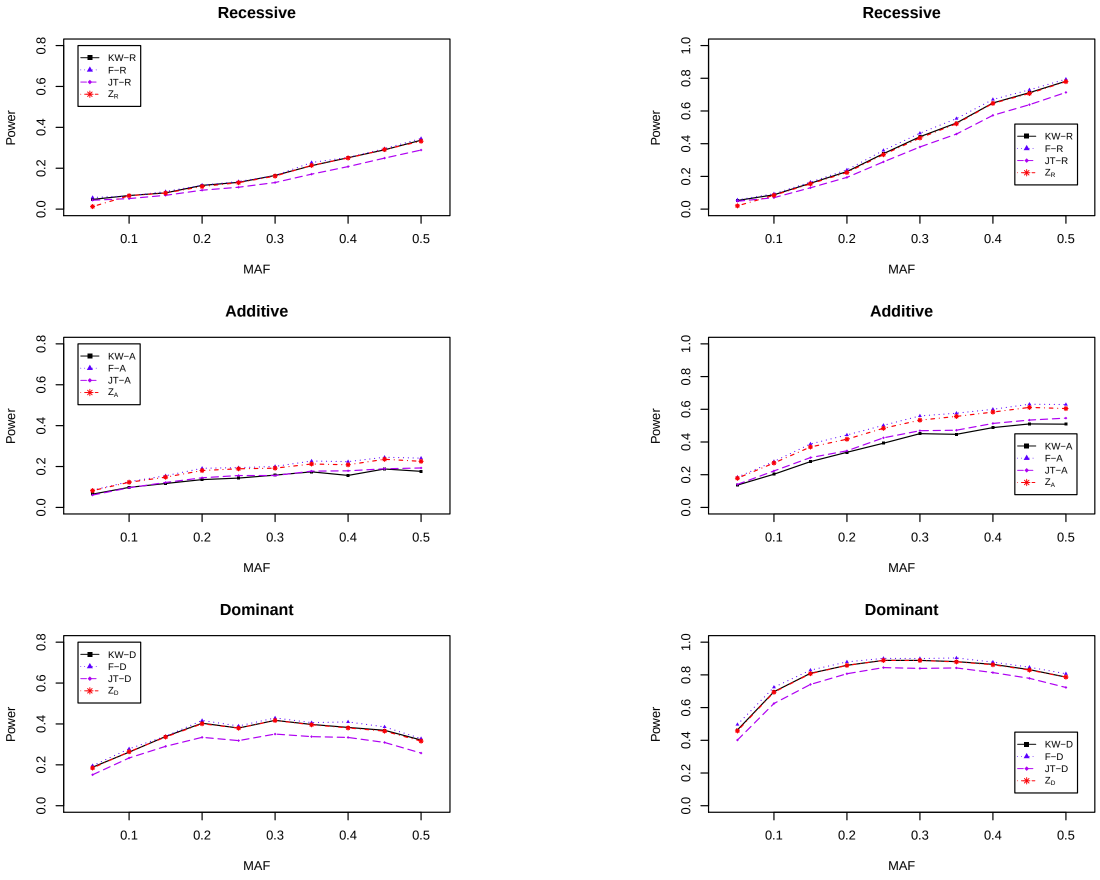

Figure S4. The empirical powers of KW-A, F-A, Z_A and MAX3 when the random error term follows the normal distribution. The first column is for $\beta_1 = \ln 1.2$ and the second column is for $\beta_1 = \ln 1.4$.

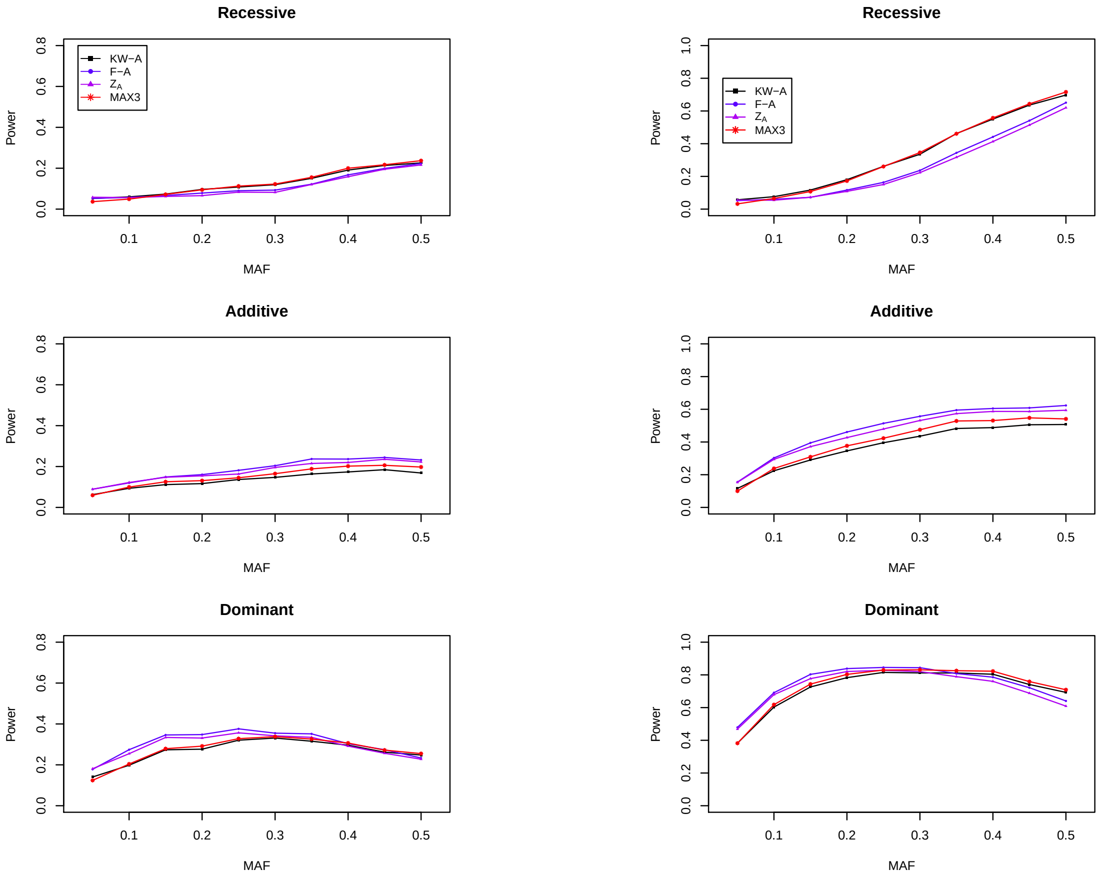

9. Simulation results for the random error term following the centralized t-distribution.

Consider the linear model $Y = \beta_0 + G\beta_1 + a \times \epsilon$, where $Y = (y_1, y_2, \cdots, y_n)^\tau$ denotes the trait value, a is a scale parameter, $G = (g_1, g_2, \cdots, g_n)^\tau$ denotes the genotype value at a single nucleotide polymorphism (SNP) locus with $g_i \in \{0, 1, 2\}$ being the count of a certain allele, and $\epsilon = (\epsilon_1, \epsilon_2, \cdots, \epsilon_n)^\tau$ denotes the random error with ϵ_i *i.i.d.* following a t-distribution with the degree of freedom b. We consider $\beta_0 = 0.5$, $\beta_1 \in \{0, \ln 1.2, \ln 1.4\}$, $a = 3$ and $b = 3$. The nominal significant level is 0.05 and 2,000 replicates are conducted. In Table S23, we report the empirical type I error rates for the sample size $n = 1500$. It shows that our proposed test can control the type I error rate. Figure S5 shows the empirical power results of KW (KW-R, KW-A, KW-D), F (F-R, F-A, F-D), JT (JT-R, JT-A,JT-D), and NRT (Z_R, Z_A, Z_D). It indicates that the proposed NRT (Z_R, Z_A, Z_D)is more powerful than the other existing test, especially under the additive model. The power results of KW-A, F-A, Z_A and MAX3 are shown in Figure S6. The results are similar to that of the tGEV error.

Table S23. The empirical type I error rates of the Kruskal-Wallis test (KW-R, KW-A, KW-D), the F test (F-R, F-A, F-D), the Jonckheere-Terpstra test (JT-R, JT-A, JT-D), the KLH and the proposed test (Z_R, Z_A, Z_D) and the proposed MAX3 when the random error term follows the centralized t-distribution.

MAF	KW-R	KW-A	KW-D	F-R	F-A	F-D	JT-R	JT-A	JT-D	KLH	Z_R	Z_A	Z_D	MAX3
0.05	0.048	0.041	0.051	0.046	0.049	0.045	0.037	0.041	0.037	0.185	0.017	0.047	0.050	0.030
0.15	0.058	0.053	0.045	0.054	0.049	0.048	0.042	0.037	0.036	0.063	0.058	0.046	0.044	0.047
0.30	0.051	0.051	0.059	0.048	0.050	0.048	0.039	0.045	0.042	0.060	0.050	0.049	0.058	0.057
0.45	0.042	0.044	0.052	0.039	0.049	0.053	0.035	0.031	0.038	0.049	0.042	0.041	0.051	0.043

Figure S5. **The empirical powers of the Kruskal-Wallis test, the F test, the Jonckheere-Terpstra test and the proposed test when the random error term follows the centralized t-distribution. The first column is for $\beta_1 = \ln 1.2$ and the second column is for $\beta_1 = \ln 1.4$.**

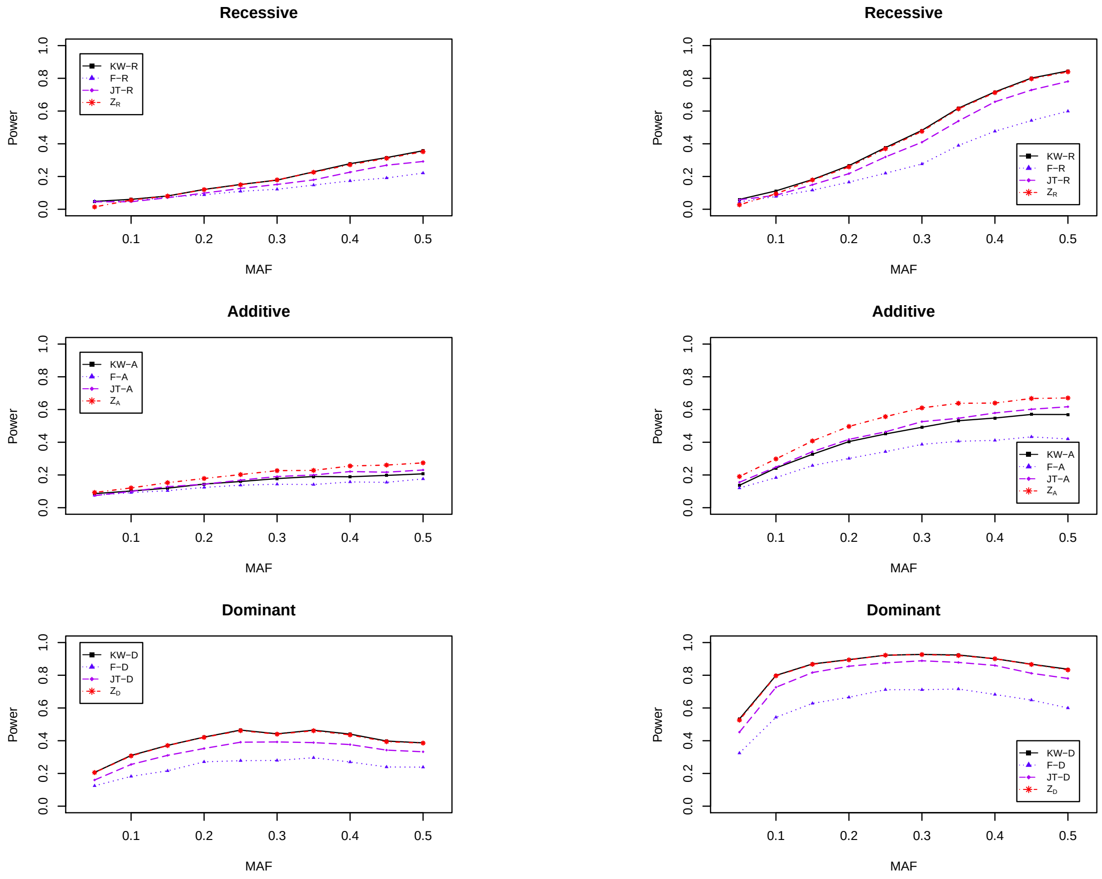

Figure S6. The empirical powers of the KW-A, F-A, Z_A and MAX3 test when the random error term follows the centralized t-distribution. The first column is for $\beta_1 = \ln 1.2$ and the second column is for $\beta_1 = \ln 1.4$.

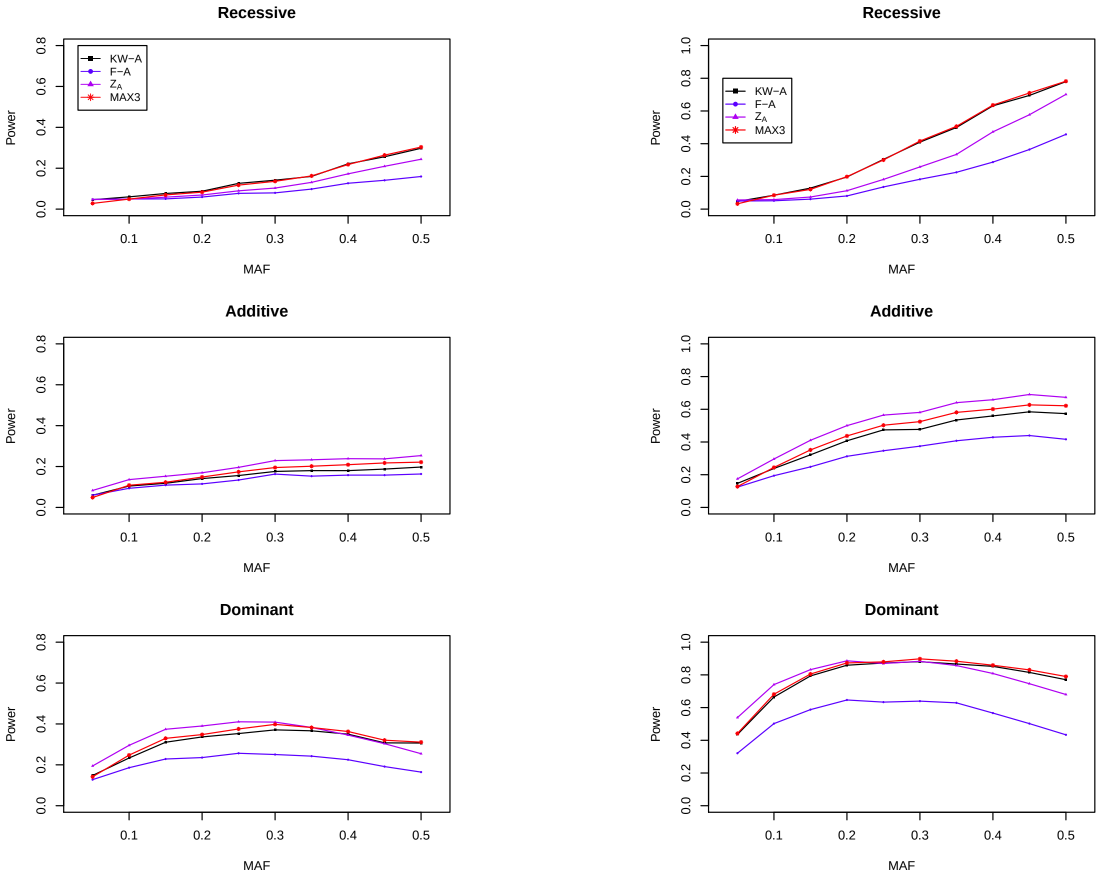

Supplement: Supplementary Information [file srep12105-s1.pdf]
